# Insulin and mTOR Pathway Regulate HDAC3-Mediated Deacetylation and Activation of PGK1

**DOI:** 10.1371/journal.pbio.1002243

**Published:** 2015-09-10

**Authors:** Shiwen Wang, Bowen Jiang, Tengfei Zhang, Lixia Liu, Yi Wang, Yiping Wang, Xiufei Chen, Huaipeng Lin, Lisha Zhou, Yukun Xia, Leilei Chen, Chen Yang, Yue Xiong, Dan Ye, Kun-Liang Guan

**Affiliations:** 1 Key Laboratory of Molecular Medicine of Ministry of Education and Institutes of Biomedical Sciences, Shanghai Medical College, College of Life Science, Fudan University, Shanghai, China; 2 Key Laboratory of Synthetic Biology, Bioinformatics Center and Laboratory of Systems Biology, Institute of Plant Physiology and Ecology, Shanghai Institutes for Biological Sciences, Chinese Academy of Sciences, Shanghai, China; 3 Lineberger Comprehensive Cancer Center, Department of Biochemistry and Biophysics, University of North Carolina at Chapel Hill, Chapel Hill, North Carolina, United States of America; 4 Department of Pharmacology and Moores Cancer Center, University of California San Diego, La Jolla, California, United States of America; Mount Sinai Hospital, CANADA

## Abstract

Phosphoglycerate kinase 1 (PGK1) catalyzes the reversible transfer of a phosphoryl group from 1, 3-bisphosphoglycerate (1, 3-BPG) to ADP, producing 3-phosphoglycerate (3-PG) and ATP. PGK1 plays a key role in coordinating glycolytic energy production with one-carbon metabolism, serine biosynthesis, and cellular redox regulation. Here, we report that PGK1 is acetylated at lysine 220 (K220), which inhibits PGK1 activity by disrupting the binding with its substrate, ADP. We have identified KAT9 and HDAC3 as the potential acetyltransferase and deacetylase, respectively, for PGK1. Insulin promotes K220 deacetylation to stimulate PGK1 activity. We show that the PI3K/AKT/mTOR pathway regulates HDAC3 S424 phosphorylation, which promotes HDAC3-PGK1 interaction and PGK1 K220 deacetylation. Our study uncovers a previously unknown mechanism for the insulin and mTOR pathway in regulation of glycolytic ATP production and cellular redox potential via HDAC3-mediated PGK1 deacetylation.

## Abstract 2

Insulin and mTOR use acetylation state to regulate phosphoglycerate kinase, a key enzyme that coordinates the balance between energy production, biosynthesis, and redox potential.

## Author Summary

Phosphoglycerate kinase (PGK1) catalyzes the reversible phosphotransfer reaction from 1, 3-bisphosphoglycerate (1, 3-BPG) to ADP to form 3-phosphoglycerate (3-PG) and ATP. By controlling ATP and 3-PG levels, PGK1 plays an important role in coordinating energy production with biosynthesis and redox balance. In contrast to the extensive investigation of the transcriptional regulation of PGK1, little is known about its post-translational regulation. Here, we report that PGK1 is acetylated at lysine 220 (K220) and this acetylation inhibits PGK1 activity by disrupting the binding with its substrate, ADP. We have identified KAT9 and HDAC3 as the acetyltransferase and deacetylase, respectively, for PGK1. Moreover, we show there is molecular crosstalk between mTOR-mediated HDAC3 S424 phosphorylation and PGK1 K220 acetylation. Our study uncovers a previously unknown mechanism for the insulin and mTOR pathway in regulating glycolytic ATP production and cellular redox potential via HDAC3-mediated PGK1 deacetylation.

## Introduction

Phosphoglycerate kinase (EC 2.7.2.3; PGK) catalyzes the reversible phosphotransfer reaction from 1, 3-bisphosphoglycerate (1, 3-BPG) to ADP to form 3-phosphoglycerate (3-PG) and ATP. The PGK-catalyzed reaction is the first ATP-yielding step of glycolysis and is essential for energy generation by the glycolytic pathway of aerobes and the fermentation of anaerobes in most living cells [1]. Besides ATP, the other product of PGK-catalyzed reaction is 3-PG, which can not only serve as a glycolytic intermediate but also be oxidized by phosphoglycerate dehydrogenase (PHGDH) to form 3-phosphohydroxypyruvate and thus enter one-carbon metabolism [2]. It is known that one-carbon metabolism involving the folate and methionine cycle integrates carbon units from amino acids, including serine and glycine, and creates various outputs, such as the maintenance of redox status by affecting glutathione biosynthesis and NADPH production, the synthesis of lipids, nucleotides, and substrate for methylation reactions [3–5]. By controlling ATP and 3-PG levels, PGK therefore plays an important role in coordinating energy production with biosynthesis and redox balance.

Regulation of PGK has been studied extensively, with research mostly focused on the transcriptional level. In yeast cells, *PGK* is one of the most highly expressed genes and accounts for approximately 5% of the total mRNA and protein [6]. *PGK* gene expression can be regulated by diverse carbon sources, with glucose induction and pyruvate suppression having been observed in yeast cells [7–9]. *PGK* gene expression is also up-regulated by oxidative stress [10]. Overexpression of PGK can suppress the apoptotic phenotypes induced by high ROS and restore normal aging of yeast cells [11]. In humans, PGK has two isoforms (PGK1 and PGK2), which share 87% identity in amino acid sequence and are structurally and functionally similar, but have different expression patterns [12–14]. *PGK1* (Gene ID: 5230) is broadly expressed in most cell types, while *PGK2* (Gene ID: 5232) is uniquely expressed in meiotic and postmeiotic spermatogenic cells [13]. *PGK1* gene as the target of the hypoxia-inducible transcriptional factor HIF-1α has been reported to be selectively up-regulated by oxidants in cultured human colon carcinoma cells [15] and hepatoblastoma cells [16,17]. In contrast to the extensive investigation on the transcriptional regulation of PGK1, little is known about its post-translational regulation.

Protein acetylation has recently been discovered as an evolutionarily conserved post-translational modification in the regulation of a wide range of cellular processes, particularly in nuclear transcription and cytoplasmic metabolism [18–20]. Together with several recent acetylome proteomic studies [21–23], more than 4,500 acetylated proteins, including human PGK1, have been identified by the mass spectrometric analyses. In this study, we investigate the regulatory mechanism and functional consequence of PGK1 acetylation.

## Results

### K220 Acetylation Inhibits PGK1 Activity via Blocking Its Binding with Substrate ADP

**Fig 1:**
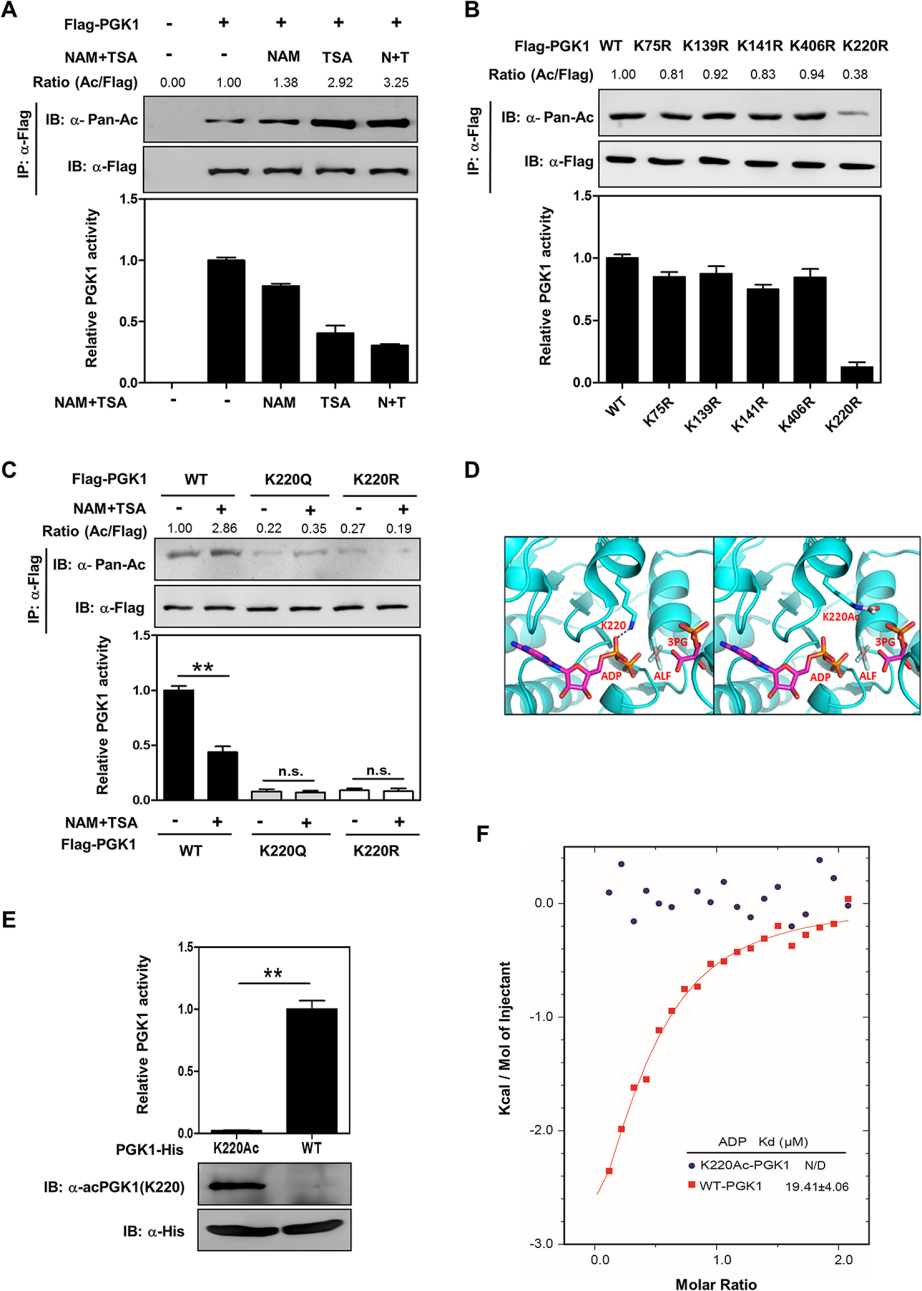
Acetylation of PGK1 K220 blocks substrate ADP binding and inhibits PGK1 activity. (**A**) Acetylation inhibits PGK1 enzyme activity. Flag-PGK1 was overexpressed in HEK293T cells followed by treatment with nicotinamide (NAM, 10 mM for 4 hr), trichostatin (TSA, 5 μM for 12 hr), or NAM and TSA (N+T). Acetylation levels and enzyme activity of Flag-bead-purified PGK1 were determined by western blot analysis and enzyme assay, respectively. Acetylation levels were blotted with a pan-anti-acetyllysine antibody (α-Ac). The catalytic activity of affinity purified PGK1 was determined and normalized to protein levels. PGK1 activity under no treatment condition was set as 1. PGK1 acetylation levels were normalized against Flag IB as indicated on the top of each lane. IB and IP denote immunoblotting and immunoprecipitation, respectively. (**B**) Mapping the major regulatory acetylation sites in PGK1. Wild-type (WT) PGK1 and the indicated mutants were each expressed in HEK293T cells. Proteins were purified by IP, and PGK1 activity was determined. Relative PGK1 acetylation levels were normalized against Flag. (**C**) K220 is a major regulatory acetylation site in PGK1. Flag-tagged wild-type and K220 mutant PGK1 were each expressed in HEK293T cells, following treatments with or without NAM (10 mM, 4 hr) and TSA (5 μM, 12 hr). Acetylation levels and enzyme activity of PGK1 were determined. (**D**) Molecular interaction between PGK1 and its substrates. As shown, PGK1 is rendered in cyan-colored illustrations, and ADP is in sticks and colored by atom type with magenta illustrations. The left panel shows that PGK1 in complex with ADP, 3-PG, and ALF (tertrafluoroaluminate) results in an active and fully closed conformation. A gray dashed line represents the hydrogen bond between K220 and ADP. 3-PG and ALF are also rendered in sticks, and colored by atom type, with carbons colored magenta. The right panel shows that the interaction between an acetylated K220 (white carbons) and ADP is disrupted. (**E**) Purification of K220 acetylated PGK1. His-tagged PGK1 and PGK1^K220ac^ were recombinantly expressed and purified from a genetically engineered *Escherichia coli*. PGK1 protein and K220 acetylation were detected with anti-His antibody as well as a site-specific anti-acetyllysine antibody [α-acPGK1(K220)]. (**F**) PGK1 K220 acetylation blocks ADP binding. The purified unacetylated PGK1 and PGK1^K220ac^ proteins were measured for their binding with substrate ADP as determined by isothermal calorimetric titration (ITC). Shown are average values with standard deviation (S.D.) of triplicated experiments. ** denotes that *p* < 0.01 for the indicated comparison; n.s. = not significant. The numerical data and statistical analysis used in the figures are included in S1 Data. Supporting information can be found in S1, S2, S3 and S4 Figs.

Previous proteomic studies have identified that PGK1 is acetylated on multiple lysine residues [18–20]. Western blotting with a pan anti-acetyl lysine antibody demonstrated that PGK1 was indeed acetylated, and its acetylation was elevated by 3.2-fold in HEK293T cells after treatment with nicotinamide (NAM), an inhibitor of the SIRT family deacetylases [24,25], and trichostatin A (TSA), an inhibitor of histone deacetylase (HDAC) I and II (Fig 1A) [26,27]. By performing enzyme activity assay in vitro, we found that the specific enzyme activity of PGK1 was decreased by as much as 63% after NAM and TSA treatment (Fig 1A), suggesting that acetylation negatively regulates PGK1 activity. Notably, the effect of TSA on increasing PGK1 acetylation and decreasing PGK1 activity was more potent than that of NAM (Figs 1A, S1A and S1B), implying that a HDAC I and/or II is the major deacetylase for PGK1.

Given that PGK1 is a highly conserved protein [28], we speculated that important regulatory sites in PGK1 targeted by acetylation might also be conserved. Sequence alignments from diverse species revealed that of twenty putative acetylated lysines identified by the different mass spectrometric analyses, fifteen lysine residues (K11, K30, K48, K86, K91, K97, K131, K146, K156, K192, K199, K264, K267, K291, K323) are not conserved, while five (K75, K139, K141, K220, K406) are invariably conserved (S2 Fig). To determine which lysine residue(s) plays a major role in the regulation of PGK1, we mutated each of the five conserved putative acetylated lysine residues in PGK1 to arginine (R) or glutamine (Q) and assayed their enzyme activity individually. The K to R mutation is often used as a deacetylation mimetic, whereas the K to Q mutation may act as a surrogate of acetylation [29]. We found that substitution at K220, but not the other four lysine residues (K75, K139, K141, and K406), by arginine substantially reduced PGK1 enzyme activity by 82% (Fig 1B), indicating K220 has an important role in controlling PGK1 activity. Moreover, both the K220R and K220Q mutants exhibited a negligible response in changing acetylation level and enzyme activity upon NAM and TSA treatment (Figs 1C and S3), re-affirming that K220 is a major acetylation site in PGK1.

PGK1 exists as a monomer comprising two nearly equal-sized N- and C-terminal domains. This extended two-domain structure is associated with large-scale “hinge-bending” conformational changes, bringing the two substrates into close proximity, with 3-PG or 1,3-BPG binding to the N-terminal domain and the nucleotide substrate ADP binding to the C-terminal domain of the enzyme [30,31]. PGK1 in complex with 3-PG, ADP, and tetrafluoroaluminate (ALF) results in a fully active and closed conformation [14]. Molecular modeling predicts that K220 acetylation disturbs PGK1’s binding with ADP (Fig 1D), suggesting that K220 acetylation may inhibit PGK1 enzyme activity by blocking the substrate ADP binding.

To test this hypothesis, we employed an expression system genetically encoding N^ε^-acetyllysine to prepare recombinant PGK1 protein that was completely acetylated on K220 in *Escherichia coli* [32,33]. Briefly, the K220 codon in PGK1 was mutated to an amber stop codon. An amber suppressor tRNA and a tRNA synthetase that could conjugate the acetyllysine to the amber tRNA suppressor were also expressed in the bacteria. Therefore, in the presence of acetyllysine added in the culture medium, the amber stop codon at the position of K220 was replaced by an acetyllysine when PGK1 was expressed in the genetically engineered *E*. *coli* strain. This expression system produced PGK1 proteins with 100% acetylation at the lysine residue 220. Moreover, we generated and verified an antibody that specifically recognizes the K220 acetylated PGK1 [α-acPGK1(K220)] (S4A, S4B and S4C Fig). The K220 acetylation of the recombinant PGK1 was confirmed by immunoblotting with the site-specific α-acPGK1(K220) antibody (Fig 1E). Consistent with the structural prediction, the recombinant PGK1^K220ac^ protein was catalytically inactive when compared to recombinant wild-type PGK1. Importantly, isothermal titration calorimetry (ITC) analysis demonstrated that the recombinant PGK1^K220ac^ protein was defective in binding with ADP (Fig 1F). These results provide direct and unequivocal biochemical evidence to support a model that K220 acetylation in PGK1 inhibits its enzymatic activity by blocking the substrate ADP binding.

### KAT9/ELP3 Is a Potential Acetyltransferase of PGK1

**Fig 2:**
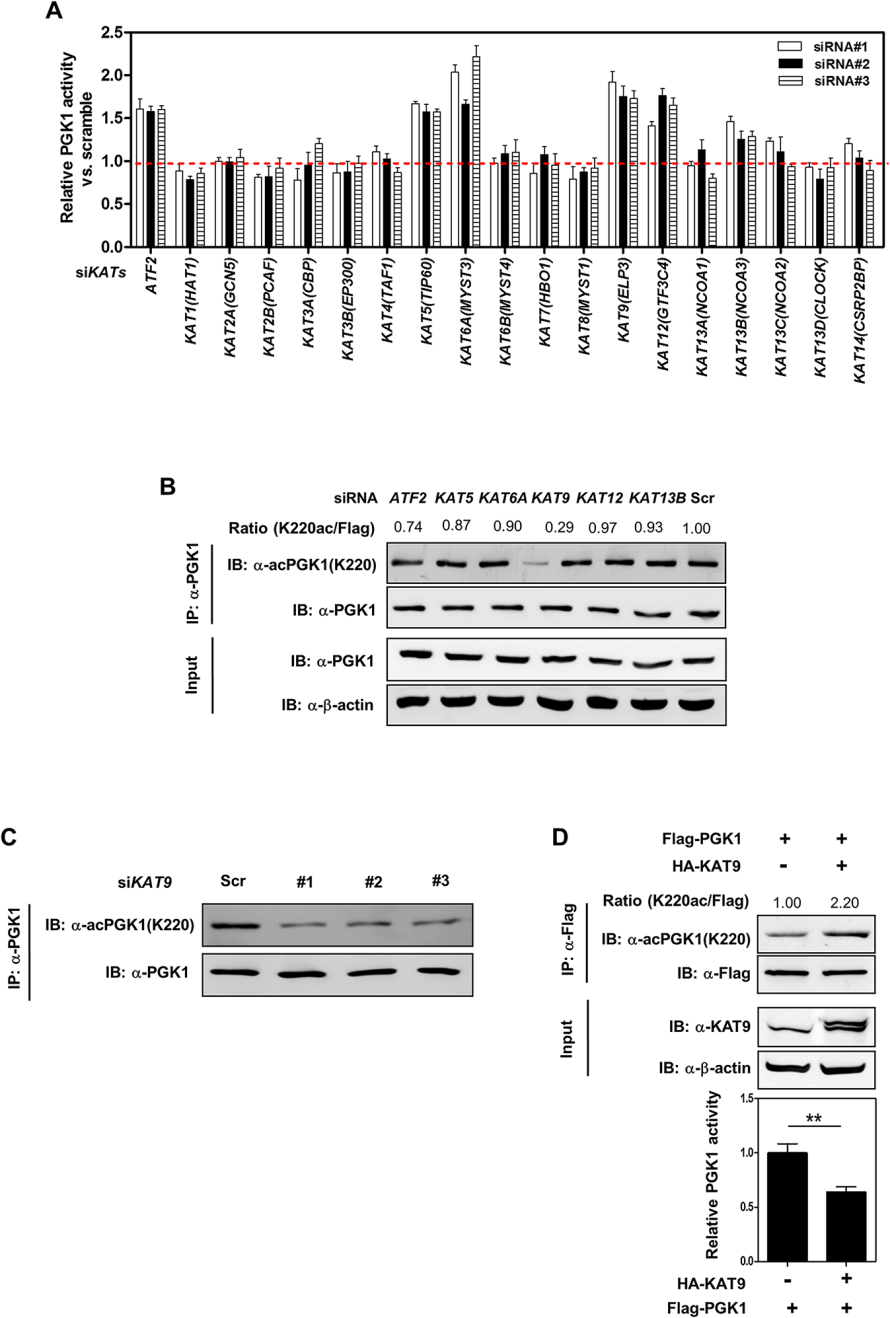
KAT9 acetylates PGK1 and inhibits PGK1 activity. (**A**) Knockdown of *ATF2*, *KAT5*, *KAT6A*, *KAT9*, *KAT12*, or *KAT13B* leads to increased enzyme activity of endogenous PGK1. HEK293T cells were transiently transfected with the indicated siRNAs. The enzyme activity of endogenous PGK1 was determined as described in “Methods and Materials.” (**B, C**) *KAT9* knockdown leads to decreased K220 acetylation of endogenous PGK1. HEK293T cells were transiently transfected with the indicated siRNAs. The protein levels and K220 acetylation of endogenous PGK1 were determined by western blot (B). Moreover, HEK293T cells were transiently transfected with three different siRNAs targeting *KAT9*, and the K220 acetylation level of endogenous PGK1 was determined by western blot (C). “Scr” means scramble. Relative PGK1 K220 acetylation levels were normalized against endogenous PGK1. (**D**) KAT9 overexpression increases PGK1 K220 acetylation and inhibits PGK1 enzyme activity. Flag-tagged PGK1 and HA-tagged KAT9 were transiently co-overexpressed in HEK293T cells, and PGK1 protein was purified by IP with Flag beads, the K220 acetylation level and enzyme activity of PGK1 were determined by western blot and enzyme assay, respectively. Relative PGK1 acetylation levels were normalized against Flag. Shown are average values with standard deviation (S.D.) of triplicated experiments. ** denotes the *p* < 0.01 for the indicated comparison; n.s. = not significant. The numerical data and statistical analysis used in the figures are included in S1 Data. Supporting information can be found in S5 Fig and S1 Table.

The acetylation state of a given protein is controlled by the action of lysine acetyltransferases (KATs) and deacetylases (KDACs), enzymes that catalyze the addition and removal, respectively, of an acetyl group from a lysine residue. To search for potential KAT(s) which are responsible for PGK1 K220 acetylation, we generated a siRNA library with three siRNAs targeting each of the 19 human *KAT* genes [34]. The knockdown efficiency of each siRNA against corresponding *KAT* genes was determined by quantitative RT-PCR (S5 Fig and S1 Table). We found that knockdown of *ATF2* (Gene ID: 1386), *KAT5* (Gene ID: 10524), *KAT6A* (Gene ID: 7994), *KAT9* (Gene ID: 55140), *KAT12* (Gene ID: 9329), or *KAT13B* (Gene ID: 8202) led to an increase in cellular PGK1 enzyme activity (Fig 2A), indicating that these KAT enzymes may play a direct or indirect role in the regulation of PGK1 activity. Among these six candidate *KAT* genes, knockdown of *KAT9* (also known as *ELP3*), but not the other five *KATs*, significantly decreased the K220 acetylation level of endogenous PGK1 without changing its protein expression in HEK293T cells (Fig 2B and 2C). In a converse experiment, co-overexpression of HA-KAT9 with Flag-PGK1 increased the PGK1 K220 acetylation level by 2.2-fold and decreased PGK1 activity by 36% (Fig 2D). Collectively, these results indicate that KAT9 is a potential acetyltransferase of PGK1.

### HDAC3 Deacetylates and Activates PGK1

**Fig 3:**
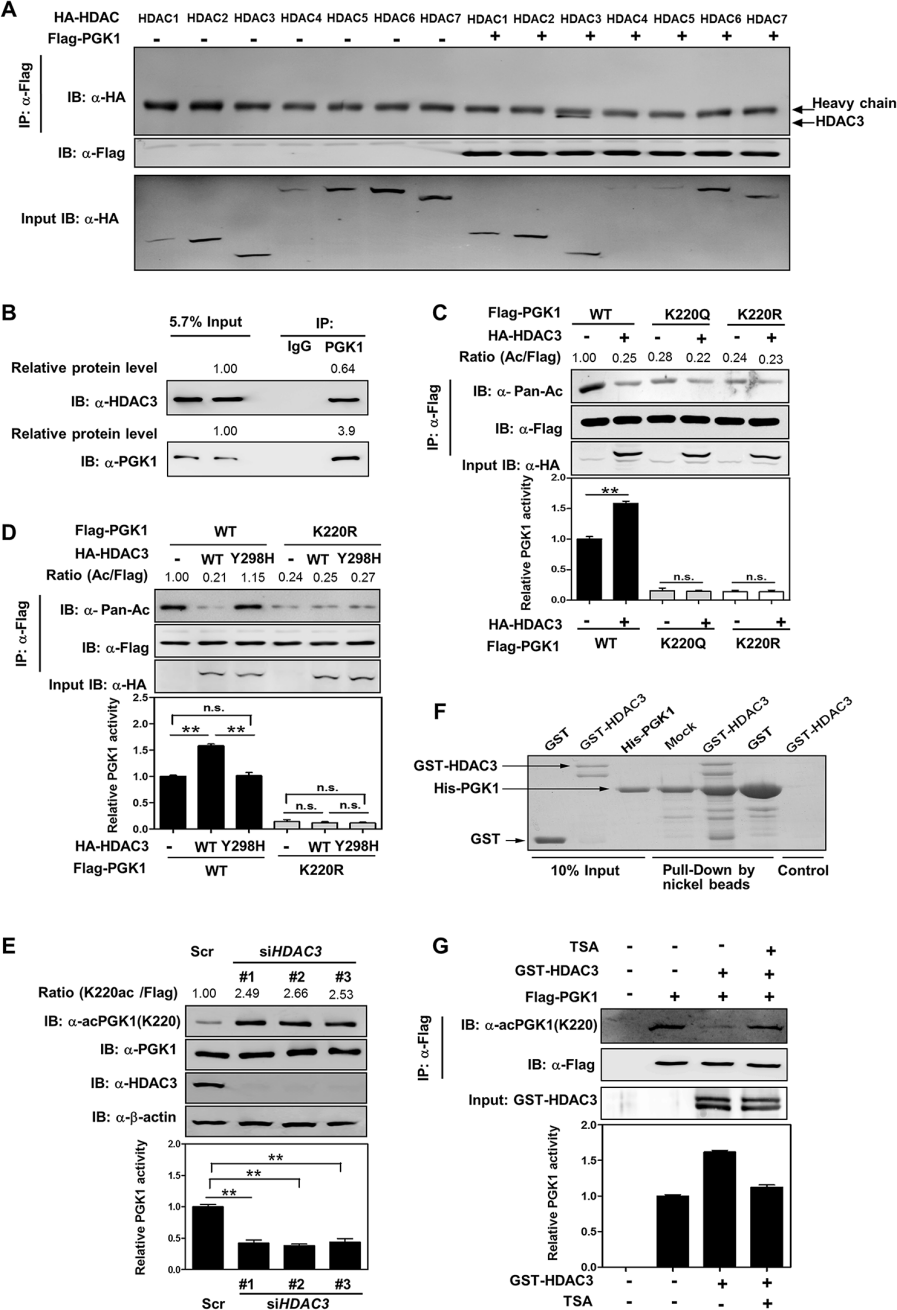
HDAC3 deacetylates and activates PGK1. (**A, B**) PGK1 interacts with HDAC3. Flag-tagged PGK1 was overexpressed in HEK293T cells together with the individual HA-tagged HDAC as indicated. PGK1 protein was purified by IP with Flag beads, following western blot to detect HDACs with a human influenza hemagglutinin (HA) antibody (A). In addition, PGK1 protein was purified by IP with PGK1 antibody, following western blot to detect its interacting endogenous HDAC3 (B). ‘5.7% input’ refers to 5.7% of total proteins for IP experiments were loaded. Based on the protein amount as determined by western blotting and chemiluminescent detection, the protein level of immunorecipitated PGK1 is 3.9 times of PGK1 input, and the protein level of co-immunorecipitated HDAC3 is 0.64 times of HDAC3 input. Thus, the IP efficiency for PGK1 is 3.9 × 5.7% = 22.2%, and the co-IP efficiency for HDAC3 is 0.64 × 5.7% = 3.6%. This indicates that ~16% (3.6%/22.2% = 16%) of endogenous HDAC3 interacting with PGK1. (**C**) HDAC3 deacetylates PGK1 mainly at the site of K220. Flag-tagged wild-type and K220 mutant PGK1 were each expressed in HEK293T cells co-expressing HA-tagged HDAC3. PGK1 proteins were purified by Flag beads, and acetylation and enzyme activity were determined by western blot and enzyme assay, respectively. Relative PGK1 acetylation levels were normalized against Flag. (**D**) PGK1 deacetylation and activation are directly associated with HDAC3 catalytic activity. Flag-tagged wild-type and K220R mutant PGK1 were each expressed in HEK293T cells co-expressing HA-tagged HDAC3 or its catalytic inactive mutant HDAC3^Y298H^. PGK1 proteins were purified by Flag beads, and acetylation and enzyme activity were determined by western blot and enzyme assay, respectively. Relative PGK1 acetylation levels were normalized against Flag. (**E**) *HDAC3* knockdown inhibits PGK1 activity via increasing PGK1 K220 acetylation. The *HDAC3* knockdown efficiency was verified by western blot using an antibody against HDAC3. The K220 acetylation level and enzyme activity of PGK1 were determined by western blot analysis and enzyme assay, respectively. ‘Scr’ means scramble. Relative PGK1 K220 acetylation levels were normalized against endogenous PGK1. (**F**) HDAC3 directly binds with PGK1. Recombinant proteins of GST-HDAC3 (30 μg) and His-PGK1 (10 μg) were purified from *E*. *coli*. In vitro pull-down assay was performed as described in S1 Text. (**G**) HDAC3 directly deacetylates PGK1. Flag-tagged PGK1 proteins were overexpressed in HEK293T cells and then purified by IP with Flag beads, following incubation with recombinant protein of GST-HDAC3 (30 μg) without or with TSA (5 μM). The K220 acetylation level and enzyme activity of PGK1 were determined by western blot and enzyme assay, respectively. Relative PGK1 acetylation levels were normalized against Flag. Shown are average values with standard deviation (S.D.) of triplicated experiments. ** denotes the *p* < 0.01 for the indicated comparison; n.s. = not significant. The numerical data and statistical analysis used in the figures are included in S1 Data.

Our earlier observation that TSA is more potent than NAM to increase PGK1 acetylation and inhibit PGK1 activity (Fig 1A) led us to search for the HDAC enzyme(s) that mediates PGK1 deacetylation. We found by binding assay that PGK1 interacted with HDAC3 (NP_003874.2), but not the other six HDACs, when co-expressed in HEK293T cells (Fig 3A). The endogenous protein interaction between HDAC3 and PGK1 was readily detected in HEK293T cells (Fig 3B). Furthermore, based on the protein amount and the immunoprecipitation efficiency, we found that ~16% of endogenous HDAC3 interacted with endogenous PGK1 (Fig 3B). Co-overexpression of HA-HDAC3 with Flag-PGK1 decreased the acetylation level of PGK1 by 75%, and increased PGK1 activity by ~1.6-fold (Fig 3C). In contrast, co-overexpression of HA-HDAC3 with the K220R/Q mutants of PGK1 did not change PGK1 acetylation or enzyme activity (Fig 3C). When PGK1 was co-overexpressed with a catalytic inactive mutant HDAC3^Y298H^ [35], neither PGK1 acetylation nor enzyme activity was altered (Fig 3D). Conversely, depletion of *HDAC3* increased the K220 acetylation level of endogenous PGK1 by >2-fold and decreased PGK1 activity by >50% in HEK293T cells (Fig 3E). More importantly, we performed in vitro pull-down assay using purified recombinant proteins of His-PGK1 and GST-HDAC3, and found that HDAC3 directly binds with PGK1 in vitro (Fig 3F). Furthermore, in vitro deacetylation assay using Flag-tagged PGK1 and GST-HDAC3 confirmed that PGK1 is a direct substrate of HDAC3 and that HDAC3-mediated K220 deacetylation increases PGK1 activity (Fig 3G).

### Insulin Promotes K220 Deacetylation to Stimulate PGK1 Activity

**Fig 4:**
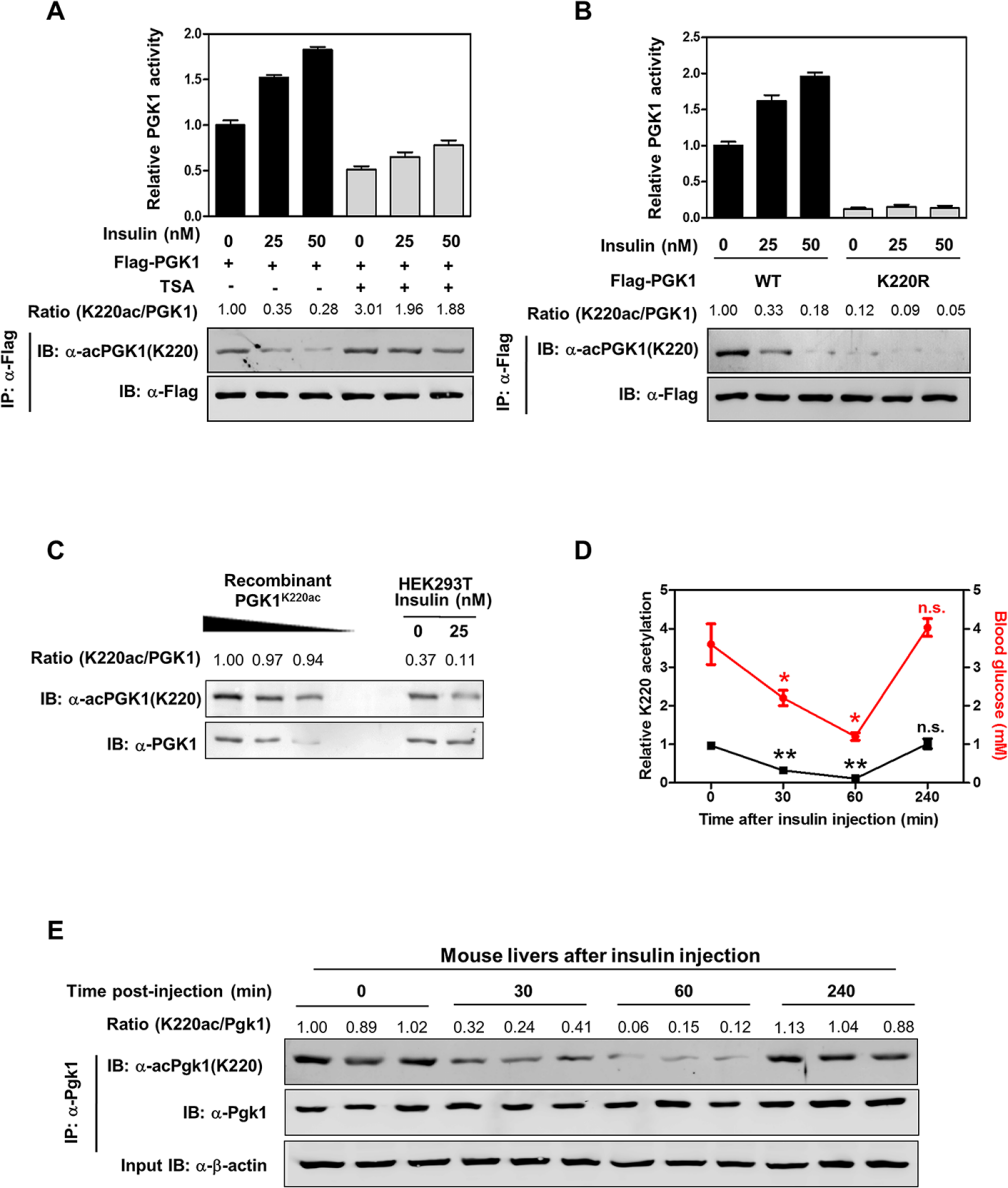
Insulin promotes K220 acetylation to stimulate PGK1 activity. (**A**) TSA treatment blocks the effect of insulin on changing PGK1 K220 acetylation and enzyme activity. Flag-tagged PGK1 was ectopically expressed in HEK293T cells treated with increased concentrations of insulin as indicated, and these cells were co-treated with or without TSA (5 μM for 12 hr). PGK1 proteins were purified by Flag beads. The K220 acetylation levels and enzyme activity were determined by western blot analysis and enzyme assay, respectively. Relative K220 acetylation levels were normalized against Flag. (**B**) K220R mutant PGK1 displays a negligible response in changing K220 acetylation and enzyme activity upon insulin treatment. Flag-tagged wild-type or K220R mutant PGK1 were transiently overexpressed in HEK293T cells treated with increased concentrations of insulin as indicated. PGK1 proteins were purified by Flag beads. The K220 acetylation levels and enzyme activity were determined by western blot analysis and enzyme assay, respectively. Relative K220 acetylation levels were normalized against Flag. (**C**) Quantification of the percentage of K220 acetylated endogenous PGK1 in HEK293T cells. Recombinant fully K220 acetylated PGK1 was loaded onto the same gel, together with endogenous PGK1 from HEK293T cells treated without or with insulin (25 nM, 2 hr). PGK1 protein and K220 acetylation were detected by western blot. Relative PGK1 K220 acetylation levels were normalized against endogenous PGK1. (**D, E**) Quantification of the percentage of K220 acetylated endogenous Pgk1 in mouse livers. Insulin (5 U/kg) was intraperitoneally injected into wild-type mice, and blood glucose levels were measured at the indicated time points after injection. Mouse livers were harvested at the indicated time points post injection, and the K220 acetylation levels of endogenous Pgk1 were determined by western blot. Relative Pgk1 K220 acetylation levels were normalized against endogenous Pgk1. Shown are average values with standard deviation (S.D.) of triplicated experiments. * denotes *p* < 0.05 and ** denotes *p* < 0.01 versus t = 0 (before injection); n.s. = not significant. The numerical data and statistical analysis used in the figures are included in S1 Data. Supporting information can be found in S6 Fig.

Previous studies have shown that *PGK1* gene expression is regulated by nutrient availability in yeast [7–9], indicating that *PGK1* may respond to energy status to maintain cellular energy homeostasis. We found that PGK1 activity was dose-dependently stimulated by insulin, an important metabolism and energy regulator, and this activation was associated with a concomitant reduction of PGK1 K220 acetylation (Fig 4A). The effect of insulin on PGK1 K220 acetylation and activity was suppressed by TSA treatment (Fig 4A). Moreover, the K220R mutant PGK1 displayed a negligible change in K220 acetylation and enzyme activity upon insulin stimulation (Fig 4B), indicating that K220 is a vital site for PGK1 deacetylation and enzymatic activation by insulin.

Next, we determined the K220 acetylation level of endogenous PGK1 in HEK293T cells. By using the recombinant PGK1^K220ac^ protein purified from *E*. *coli* as the standard, we found that ~37% of endogenous PGK1 was acetylated at K220, and that insulin treatment decreased PGK1 acetylation to 11% (Fig 4C). The fact that a significant fraction of endogenous PGK1 undergoes deacetylation upon insulin treatment suggests that K220 acetylation plays a physiologically relevant role in PGK1 regulation.

Furthermore, we assessed PGK1 K220 acetylation in mouse tissues after insulin injection. As expected, intraperitoneal injection of insulin (5 U/kg) resulted in a transient drop in blood glucose levels (Fig 4D). Interestingly, the K220 acetylation level of endogenous Pgk1 in mouse livers was significantly (*p* < 0.01) decreased and bottomed at 60 min after insulin injection, followed by a period of recovery in parallel with the blood glucose levels (Fig 4D and 4E). Similarly, Pgk1 K220 acetylation was dynamically changed in mouse kidneys after insulin injection (S6 Fig). Together, these findings suggest that K220 acetylation plays a signaling role in regulating PGK1 function both in cultured cells and in mouse tissues upon insulin treatment.

### Insulin Decreases K220 Acetylation by Enhancing PGK1-HDAC3 Association

We next set out to investigate how insulin signal regulates PGK1 K220 acetylation and activity. We found that insulin signal did not change *KAT9* gene expression in HEK293T cells (S7A Fig). The PGK1-KAT9 protein association was readily detected in cells co-overexpressing Flag-PGK1 and HA-KAT9, leading to an increase in PGK1 K220 acetylation (S7B Fig). However, neither the KAT9-PGK1 protein interaction nor PGK1 K220 acetylation was affected by insulin treatment in cells co-overexpressing PGK1 and KAT9 (S7B Fig). These findings suggest that KAT9 may not contribute to insulin-regulated PGK1 acetylation.

**Fig 5:**
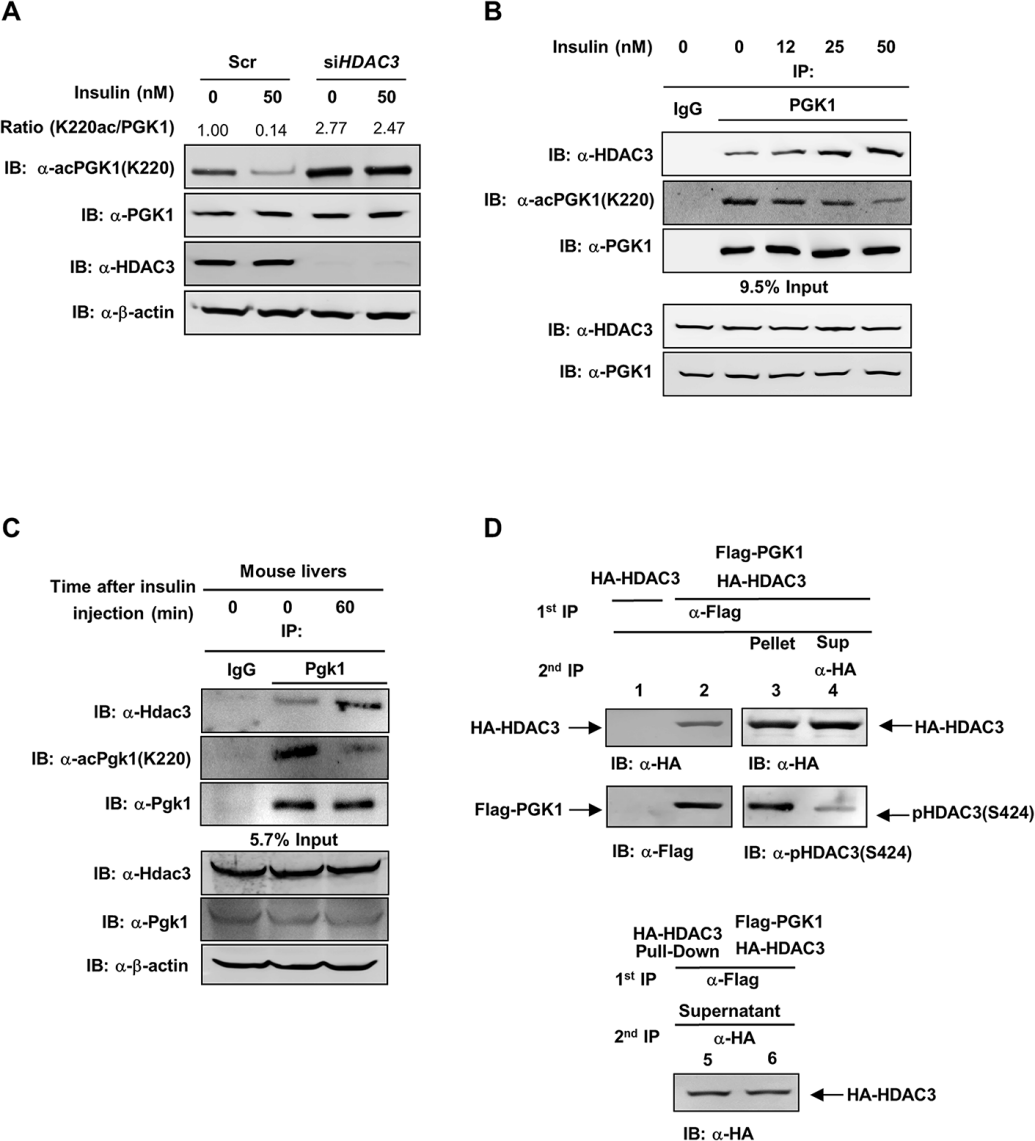
S424 Phosphorylation of HDAC3 promotes its binding with PGK1. (**A**) *HDAC3* knockdown prevents the effect of insulin on changing PGK1 K220 acetylation and enzyme activity. HEK293T cells were transiently transfected with siRNAs against *HDAC3*. At 48 hr post transfection, cells were treated with 50 nM insulin for 2 hr. “Scr” denotes scramble siRNA control. The K220 acetylation level of endogenous PGK1 was determined by western blot, and then normalized against endogenous PGK1. (**B**) Insulin enhances PGK1 interaction with HDAC3. HEK293T cells were treated with insulin at the indicated concentrations for 2 hr. PGK1 protein was immunoprecipitated and followed by western blot to detect the K220 acetylation and interaction with HDAC3. The phrase “9.5% input” refers to the fact that 9.5% of total proteins for IP experiments were loaded. (**C**) Insulin increases Pgk1 K220 acetylation and interaction with Hdac3 in mouse livers. Insulin (5 U/kg) was intraperitoneally injected into wild-type mice. At the indicated time points post injection, mouse livers were harvested, and the Pgk1 K220 acetylation and interaction with Hdac3 were determined by western-blot. The phrase “5.7% input” refers to the fact that 5.7% of total proteins for IP experiments were loaded. (**D**) Ser424 phosphorylated HDAC3 preferentially interacts with PGK1. HA-HDAC3 was transiently transfected with or without Flag-PGK1 into HEK293T cells. Cell lysates were immunoprecipitated with Flag antibody as indicated by first IP Ppt. The remaining supernatant (Sup) was precipitated with HA antibody (second IP). An equal amount of HA-HDAC3 from the first Flag-PGK1 co-IP or the second HA-HDAC3 IP was used to detect Ser424 phosphorylation with the phosphoSer424 antibody (as indicated by P-HDAC3(S424)). Supporting information can be found in S7, S8 and S9 Figs.

On the other hand, we observed that the effect of insulin on reducing the K220 acetylation level of endogenous PGK1 was diminished in HEK293T cells when *HDAC3* was depleted by siRNA (Fig 5A). Insulin treatment did not change *HDAC3* gene expression in HEK293T cells (S8 Fig), but could enhance endogenous PGK1-HDAC3 association and decrease PGK1 K220 acetylation in a dose-dependent manner (Fig 5B). In agreement, insulin injection reduced Pgk1 K220 acetylation and increased Pgk1-Hdac3 interaction in mouse livers (Fig 5C). Together, these results suggest that insulin promotes PGK1 K220 deacetylation likely through enhancing PGK1-HDAC3 association.

### S424 Phosphorylation of HDAC3 Promotes Its Binding with PGK1

Recent studies have identified HDAC3 as a phosphorylated protein, with Ser424 being one of the key phosphorylation sites [36,37]. Protein kinase CK2 has been reported to be responsible for HDAC3 S424 phosphorylation [38]. Mutation of serine (S) 424 to alanine (A) (mimics dephosphorylation) inhibits HDAC3 enzyme activity without affecting its expression or subcellular localization [38]. In accord, we found that the HDAC3^S424A^ mutant displayed a ~50% reduction in enzyme activity (S9 Fig). Strikingly, we observed that HDAC3^S424A^ mutant exhibited impaired association with endogenous PGK1 in HEK293T cells (S9 Fig), suggesting that Ser424 phosphorylation is critical for not only the deacetylase activity of HDAC3 but also its interaction with PGK1. To provide further evidence to support this notion, we transfected HEK293T cells singularly with plasmid expressing Flag-PGK1 or with plasmids co-expressing Flag-PGK1 and HA-HDAC3. Lysates from transfected cells were first immunoprecipitated with Flag antibody and then the remaining supernatant was precipitated with HA antibody. We found that HA-HDAC3 was co-precipitated with Flag-PGK1 (Fig 5D). Importantly, HA-HDAC3 in the PGK1 immune complex was highly phosphorylated on Ser424, whereas the free HDAC3 was weakly phosphorylated. These results suggest that Ser424 phosphorylation of HDAC3 may enhance its interaction with PGK1.

### The mTOR Pathway Regulates HDAC3 S424 Phosphorylation and PGK1 K220 Acetylation

**Fig 6:**
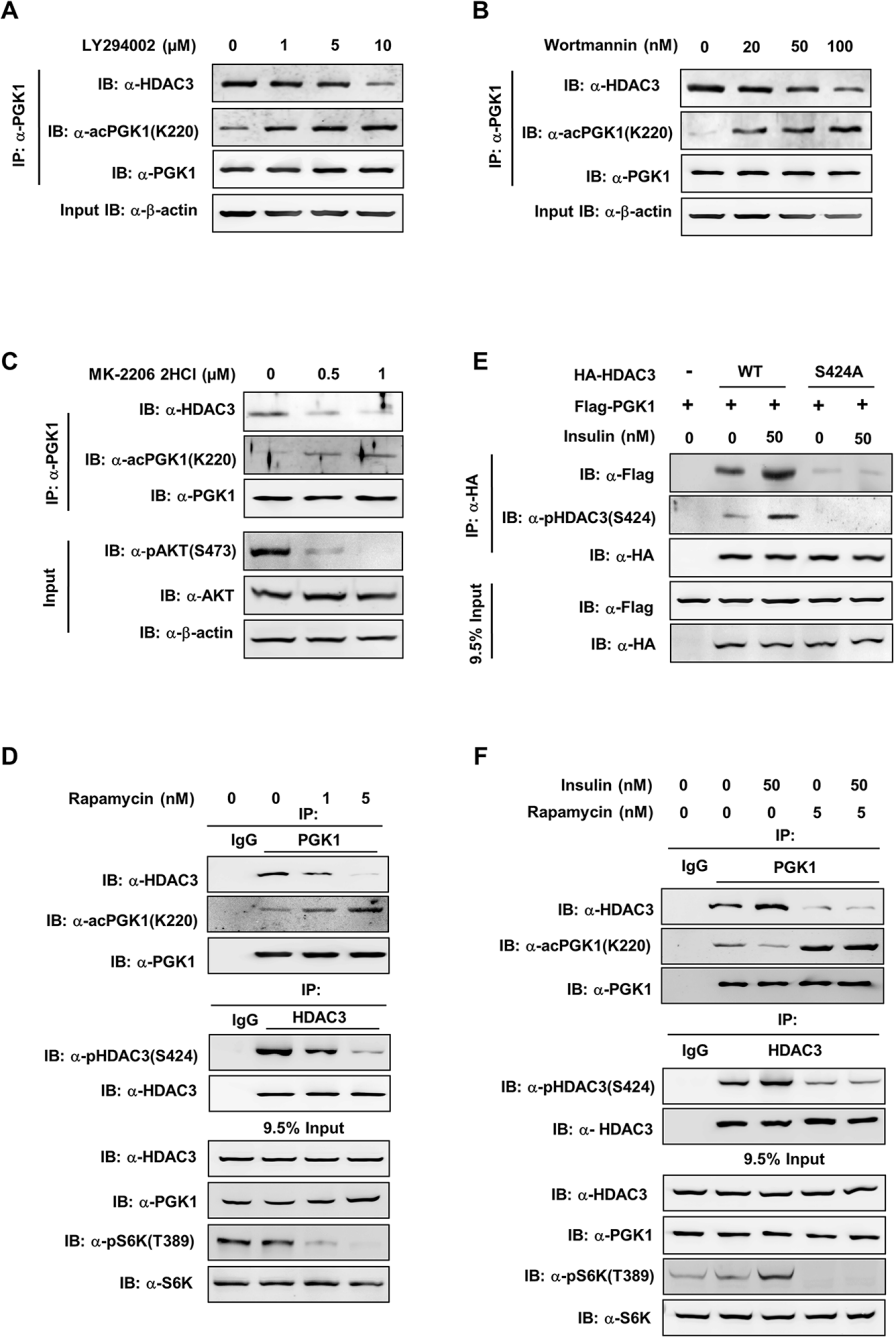
The mTOR pathway regulates HDAC3 S424 phosphorylation and PGK1 K220 acetylation. (**A, B**) Inhibition of PI3K increases the K220 acetylation level of endogenous PGK1. HEK293T cells were treated with two PI3K inhibitors, LY294002 (A) and Wortmannin (B), at the indicated concentrations for 4 hr and 2 hr, respectively. PGK1 protein was purified by IP with PGK1 antibody, following western blot to detect the K220 acetylation level of endogenous PGK1 and its interacting endogenous HDAC3. (**C**) Inhibition of AKT increases the K220 acetylation level of endogenous PGK1. HEK293T cells were treated with MK-2206 2HCI (a specific AKT inhibitor) at the indicated concentrations for 2 hr. PGK1 protein was purified by IP with PGK1 antibody, following western blot to detect the K220 acetylation level of endogenous PGK1 and its interacting endogenous HDAC3. AKT protein and AKT S473 phosphorylation levels were detected with anti-AKT antibody as well as anti-pAKT(S473) antibody. (**D**) Inhibition of mTOR decreases HDAC3 Ser424 phosphorylation and increases PGK1 K220 acetylation. HEK293T cells were treated with Rapamycin for 2 hr. Endogenous HDAC3 and PGK1 were immunopurified with HDAC3 and PGK1 antibodies, respectively, followed by western blotting to detect HDAC3 Ser424 phosphorylation and PGK1 K220 acetylation. S6K protein and S6K T389 phosphorylation levels were detected with anti-S6K antibody as well as anti-pS6K(T389) antibody. The phrase “9.5% input” refers to the fact that 9.5% of total proteins for IP experiments were loaded. (**E**) HDAC3 Ser424 is required for insulin to promote HDAC3-PGK1 interaction. Flag-PGK1 was co-overexpressed with HA-tagged wild-type or S424A mutant HDAC3 in HEK293T cells. The transfected cells were treated with insulin (0 or 50 nM, 2 hr). HA-HDAC3 protein was purified by HA beads and the co-precipitated Flag-PGK1 was detected by western blot. The phrase ‘”9.5% input” refers to the fact that 9.5% of total proteins for IP experiments were loaded. (**F**) Rapamycin treatment diminishes the effect of insulin on changing HDAC3 S424 phosphorylation, HDAC3-PGK1 association, and PGK1 K220 acetylation. HEK293T cells were treated with Rapamycin (5 nM, 2 hr) prior to insulin treatment (50 nM, 2 hr). Endogenous proteins of PGK1 and HDAC3 were purified by IP with PGK1 and HDAC3 antibodies. Western blot was performed to detect HDAC3-PGK1 interaction, and the indicated proteins and their phosphorylation. The phrase “9.5% input” refers to the fact that 9.5% of total proteins for IP experiments were loaded. Supporting information can be found in S10 Fig and S11 Fig.

Previous studies have shown that HDAC3 phosphorylation is regulated by phosphoinositide-3-kinase/AKT (PI3K/AKT) pathway [39]. In addition, HDAC3 has also been identified as a downstream target of mTOR [36,37]. This led us to hypothesize that the PI3K/AKT/mTOR signaling pathway may regulate HDAC3 S424 phosphorylation and PGK1 K220 acetylation in response to insulin. To test this hypothesis, we treated HEK293T cells co-overexpressing Flag-PGK1 and HA-HDAC3 with either LY294002 or Wortmannin, two specific PI3K inhibitors. As expected, either PI3K inhibitor profoundly attenuated AKT S473 phosphorylation (S10A and S10B Fig). Notably, both LY294002 and Wortmannin dose-dependently increased K220 acetylation of ectopically expressed Flag-PGK1 and decreased the interaction between ectopically expressed PGK1 and HDAC3 (S10A and S10B Fig). In accord, both LY294002 and Wortmannin dose-dependently increased K220 acetylation of endogenous PGK1 and decreased endogenous PGK1-HDAC3 association in HEK293T cells (Fig 6A and 6B). In addition, MK-2206 2HCI, a specific AKT inhibitor, produced similar effects on changing PGK1 K220 acetylation and PGK1-HDAC3 association as the PI3K inhibitor LY294002 or Wortmannin (Fig 6C). Furthermore, we observed that the mTOR inhibitor Rapamycin profoundly attenuated S6K T389 phosphorylation as expected, reduced HDAC3 Ser424 phosphorylation, impaired endogenous PGK1-HDAC3 interaction, and increased PGK1 K220 acetylation (Fig 6D). Collectively, these findings demonstrate that the PI3K/AKT/mTOR pathway can regulate PGK1 K220 acetylation, possibly through controlling HDAC3 S424 phosphorylation and HDAC3–PGK1 protein interaction.

The PI3K/AKT/mTOR pathway is regulated by a wide variety of cellular signals, including insulin [40]. We then studied the role of the PI3K/AKT/mTOR pathway in mediating insulin signal to control PGK1 K220 acetylation. As shown, insulin increased HDAC3 Ser424 phosphorylation and enhanced the interaction between ectopically expressed proteins of HDAC3 and PGK1 (Fig 6E). The dephosphorylation-mimicking HDAC3^S424A^ mutant exhibited weak binding to PGK1 even upon insulin treatment (Fig 6E), supporting the notion that HDAC3 S424 is a key site for regulating HDAC3-PGK1 association upon insulin stimulation. Rapamycin treatment decreased HDAC3 Ser424 phosphorylation, impaired protein interaction between endogenous HDAC3 and PGK1, and increased PGK1 K220 acetylation (Fig 6F). Moreover, rapamycin abolished the effect of insulin on changing HDAC3 Ser424 phosphorylation, HDAC3-PGK1 association, and PGK1 K220 acetylation (Fig 6F). Besides insulin, other growth factors, such as EGF, may also enhance the PI3K/mTOR pathway [41]. We found that EGF stimulation increased ERK1/2 phosphorylation, but not S6K T389 phosphorylation, in HEK293T cells, suggesting that EGF cannot potently activate mTOR in our experiment. As a result, EGF treatment did not change HDAC3 S424 phosphorylation and PGK1 K220 acetylation (S11 Fig). Collectively, these data suggest that the mTOR pathway regulates PGK1 K220 acetylation through controlling HDAC3 S424 phosphorylation.

### PGK1 K220 Acetylation Regulates Glycolytic ATP Production, Glucose Metabolism, and Redox Status

**Fig 7:**
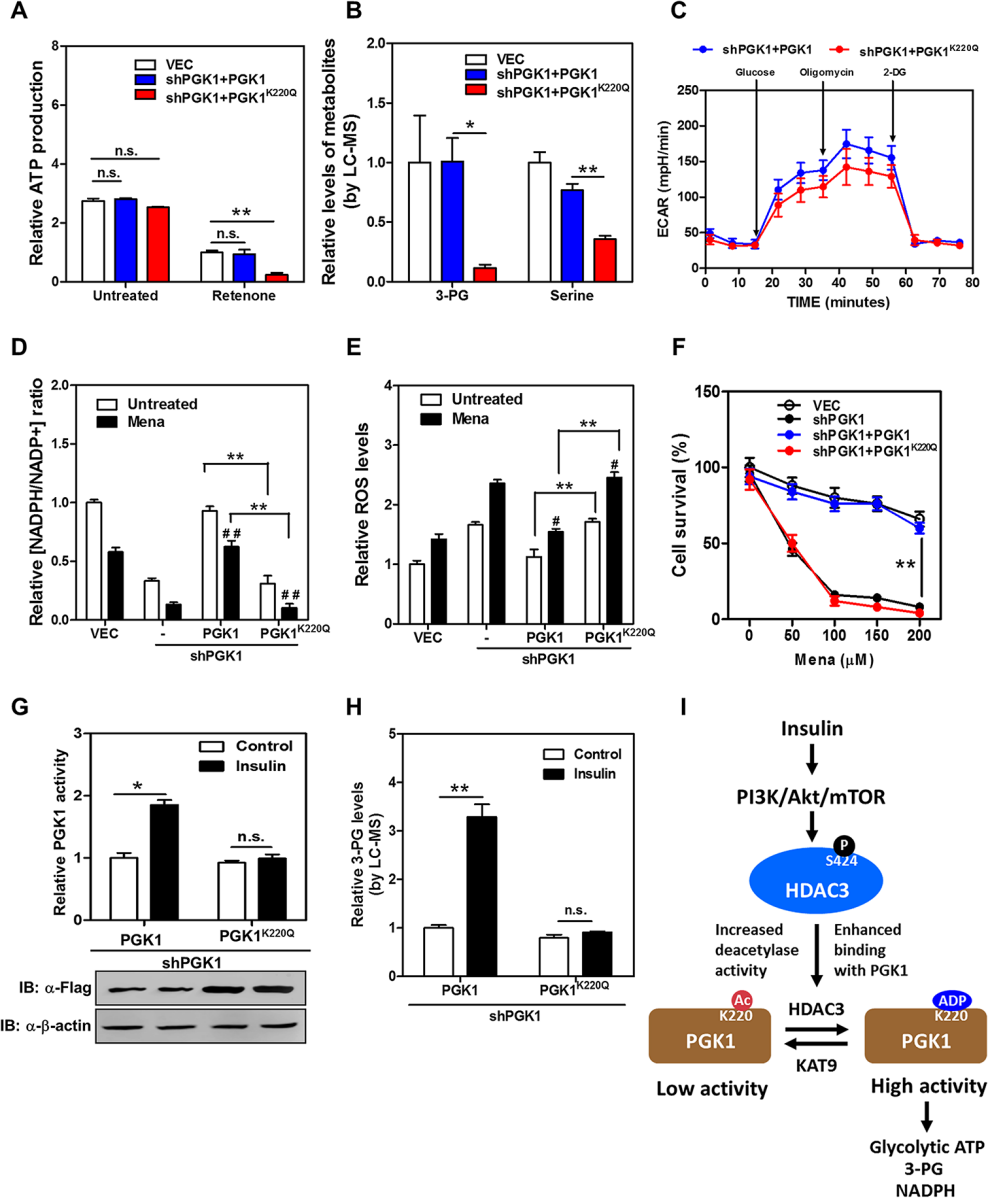
PGK1 K220 acetylation regulates glycolytic ATP production, glucose metabolism, and redox state of cells. (**A**) K220Q mutant PGK1 put-back cells display decreased glycolytic ATP production. Stable HEK293T cells were treated without or with Rotenone (100 nM for 6 hr), following measurement of cellular ATP production as described in S1 Text. The glycolytic ATP production in rescued cells expressing the empty vector (VEC, white column), wild-type PGK1 (blue column), and K220Q mutant PGK1 (red column) was compared. (**B**) K220Q mutant PGK1 put-back cells display decreased levels of 3-PG and serine. Stable HEK293T cells were rinsed with 1 ml ice-cold PBS for less than 1 min and quenched by using 80% (v/v) pre-cold (-80°C) methanol, following the standard LC-MS method to determine intracellular levels of 3-PG and serine as described in S1 Text. (**C**) K220Q mutant PGK1 put-back cells exhibit impaired glycolytic function. ECAR assay was performed in stable HEK293T cells using a Seahorse Bioscience XFe 96 analyzer system as described in S1 Text. The glycolytic function of rescued cells expressing wild-type PGK1 (blue circle) and K220Q mutant PGK1 (red circle) was compared. (**D**) K220Q mutant PGK1 put-back cells exhibit reduced NADPH/NADP^+^ ratio. Stable HEK293T cells were treated without or with menadione (50 μM for 30 min), and the ratio of NADPH/NADP^+^ was determined by enzymatic analysis as described in S1 Text. (**E**) K220Q mutant PGK1 put-back cells exhibit higher ROS production. Stable HEK293T cells were treated without or with menadione (50 μM for 30 min), and ROS accumulation was determined by using a fluorescent dye as described in S1 Text. (**F**) K220Q mutant PGK1 put-back cells show higher susceptibility to ROS-induced cell death. Stable HEK293T cells were treated with menadione at the indicated concentrations for 3 hr. The viability of cells without or with *PGK1* knockdown (white or black circle), and cells with *PGK1* knockdown and rescued by wild-type or K220Q mutant PGK1 (blue or red circle) was examined by counting the remaining adherent cells using Trypan Blue. (**G, H**) K220Q mutant PGK1 put-back cells display a negligible response in changing enzyme activity upon insulin treatment. Stable HEK293T cells were generated in which endogenous *PGK1* was depleted and the acetylated mimic K220Q mutant was re-introduced at a higher protein level in order to reach an equivalent PGK1 enzyme activity between the rescued cells expressing wild-type and K220Q mutant PGK1. These stable cells were treated without or with insulin (50 nM for 2 hr), following measurement of PGK1 activity (G) and relative 3-PG levels (H) as described in “Methods and Materials” and S1 Text. (**I**) A working model depicting that the PI3K/AKT/mTOR pathway regulates HDAC3 Ser424 phosphorylation, which enhances the protein interaction between HDAC3 and PGK1 and decreases PGK1 K220 acetylation, and that HDAC3-dependent regulation of PGK1 K220 acetylation modulates the production of glycolytic ATP and 3-PG in the cell upon insulin stimulation. In A, B, D, E, F, G, and H, shown are average values with standard deviation (S.D.) of triplicated independent experiments. In C, shown are average values with standard error of the mean (S.E.M.) of triplicated experiments. * denotes *p* < 0.05 and ** denotes *p* < 0.01 for the indicated comparisons; ^#^ denotes *p* < 0.05 and ^# #^ denotes *p* < 0.01 versus untreated group; n.s. = not significant. The numerical data and statistical analysis used in the figures are included in S1 Data. Supporting information can be found in S12, S13, S14, S15, S16, and S17 Figs.

To address the physiological significance of PGK1 regulation by K220 acetylation, we generated stable HEK293T cells in which endogenous PGK1 was depleted, and wild-type or K220 mutant PGK1 was re-introduced at a comparable level (knockdown and put-back, S12 Fig). We found that ATP production did not differ between wild-type and PGK1^K220Q^ mutant put-back cells (Fig 7A). We then treated these stable cells with rotenone, a chemical which inhibits the complex I of the respiratory chain and thus mitochondrial ATP production [42,43]. A short-term treatment with rotenone greatly decreased cellular ATP production without inducing substantial cell death (S13 Fig). Notably, PGK1^K220Q^ mutant put-back cells displayed a remarkable reduction (by 76%; *p* < 0.01) in glycolytic ATP production compared to wild-type rescued cells (Fig 7A). Liquid chromatography-mass spectrometry (LC-MS) analysis demonstrated that the level of 3-PG, which is the product of PGK1 catalysis, was dramatically reduced, by 89% (*p* < 0.05), in PGK1^K220Q^ mutant put-back cells compared to wild-type rescued cells (Fig 7B). 3-PG can be oxidized to 3-phosphohydroxypyruvate for de novo (that is originated from glucose) serine biosynthesis [44]. In accord with reduced 3-PG, the serine level was significantly decreased (by 64%; *p* < 0.01) in PGK1^K220Q^ mutant put-back cells (Fig 7B). Moreover, extracellular acidification rate (ECAR) analysis revealed that several parameters reflecting the glycolytic function, such as glycolysis, glycolytic capacity, and glycolytic reserve, were lower in PGK1^K220^ mutant put-back cells than wild-type rescued cells (Figs 7C and S14). Glucose consumption was significantly decreased (~60% less; *p* < 0.01), while glycogen storage was significantly increased (by ~2.2-fold; *p* < 0.01) in the PGK1^K220Q^ mutant put-back cells when compared to wild-type rescued cells (S15A and S15B Fig). These results suggest that K220 acetylation plays an important role in regulating PGK1 activity and/or function to modulate glycolytic ATP production and glucose metabolism.

We also observed that stable knockdown of *PGK1* led to a ~65% reduction in the NADPH/NADP^+^ ratio in HEK293T cells (Fig 7D), suggesting that PGK1 is an important contributor to NADPH pools besides its well-known role in glycolytic ATP production. Moreover, higher ROS production was detected in *PGK1* knockdown cells subjected to menadione, a quinone compound that induces the production of superoxide radicals (Fig 7E). ROS has been extensively implicated in signaling cascades, which function as important cell survival mechanisms in response to oxidative stress [45]. We found that *PGK1* knockdown cells exhibited higher levels of cleaved PARP, an indicator of apoptosis (S16A Fig), as well as higher levels of p38 MAPK phosphorylation, a stress-responsive kinase (S16B Fig). Importantly, re-expression of wild-type PGK1, but not the acetylated mimetic K220Q mutant, restored the NADPH/NADP^+^ ratio and suppressed ROS production in *PGK1* knockdown cells subjected to menadione (Fig 7D and 7E). Putting-back wild-type PGK1, but not the K220Q mutant, reduced the levels of cleaved PARP and p38 MAPK phosphorylation in *PGK1* knockdown cells when subjected to menadione (S16A and S16B Fig). As a result, *PGK1* knockdown cells exhibited a higher incidence of cell death in response to menadione, and re-expression of wild-type PGK1, but not the K220Q mutant, could rescue cells from menadione-induced cell death (Figs 7F and S17). These results suggest that K220 acetylation plays an important role in regulating PGK1 activity/function to modulate NADPH redox and cellular oxidative response.

To further illustrate the role of K220 acetylation in controlling glucose metabolism and redox potential in the cell, we generated *PGK1* knockdown and put-back stable HEK293T cells, in which endogenous *PGK1* was depleted and the K220Q mutant was re-introduced at a higher protein level in order to reach an equivalent PGK1 enzyme activity between the cells re-expressing wild-type and K220Q mutant PGK1 (Fig 7G). By monitoring the reduction of NADH, we found that the activity of wild-type PGK1 was significantly (*p* < 0.05) stimulated by insulin treatment (Fig 7G), leading to a remarkable increase of 3-PG (by 3.2-fold; *p* < 0.01) (Fig 7H). In contrast, cells with PGK1^K220Q^ mutant put-back cells displayed a negligible change in PGK1 enzyme activity and 3-PG upon insulin stimulation (Fig 7G and 7H). These findings strongly support the notion that K220 acetylation plays an important role in regulating PGK1 activity/function in cells upon physiological stimulus, such as insulin.

## Discussion

### K220 Acetylation of PGK1 Regulates Substrate ADP Binding

The current study uncovers a biochemical mechanism for how acetylation controls the activity/function of PGK1. We have identified KAT9 and HDAC3 as the potential acetyltransferase and major deacetylase of PGK1, respectively. To the best of our knowledge, we show for the first time that acetylation of PGK1 plays an important role in modulating glycolytic energy production and cellular oxidative response.

We have identified K220 as an important regulatory acetylation site within the PGK1 protein. Human PGK1 exists as a monomer containing two nearly equal-sized domains corresponding to the N- and C-termini of the protein [30]. 1, 3-BPG binds to the N-terminal domain whereas ADP binds to the C-terminal domain of PGK1 [46]. Though the binding of either substrate 1,3-BPG or ADP triggers a conformational change, only through the binding of both substrates does domain closure occur, bringing the two substrates into the proper vicinity for phosphotransfer [47,48]. Of note, K220 locates in the C-terminus of PGK1 and interacts with the nucleotide substrate ADP [48,49]. Our data show that acetylation mimetic K220Q substitution significantly reduces PGK1 catalysis. This is best illustrated by the observation that the recombinant PGK1^K220ac^ protein is defective in ADP binding, implying that neutralization of the positive charge of K220 by acetylation may disrupt ADP binding and thus inhibit PGK1 catalysis. It has to be noted that both acetylation mimetic K220Q substitution and deacetylation mimetic K220R substitution can significantly reduce PGK1 activity. This raises another possibility, that is, the steric hindrance that occurs when K220 is mutated to Q or R, and this will generate different interaction force and/or steric hindrance, thereby abolishing ADP binding and inhibiting PGK1 catalysis.

### Crosstalk between HDAC3 Phosphorylation and PGK1 Acetylation Links Extracellular Physiologic Stimuli to Intracellular Metabolic Flux

Our study has provided novel insights into the role of K220 acetylation in controlling PGK1 activity/function in response to insulin. To the best of our knowledge, this is the first evidence linking the regulation of PGK1 to mTOR downstream of PI3K/AKT and insulin. In addition, we show a molecular crosstalk between mTOR-mediated HDAC3 S424 phosphorylation and PGK1 K220 acetylation.

We have identified HDAC3 as the major deacetylase of PGK1. HDAC3 can form multi-protein complexes with the co-repressors SMRT and N-CoR and deacetylates histones, thereby regulating the transcription of a plethora of genes [50,51]. In addition, many non-histone substrates of HDAC3 have been identified, including the NF-ƘB protein RelA [52], sex-determining region Y (SRY, a master regulator of testis organogenesis) [53], and several transcription factors such as p53 [54], myocyte enhancer factor-2 (Mef2) [55], and glial cell missing (GCMa) [56]. Very recently, it was reported that HDAC3 deacetylates methionine adenosyltransferase IIα (MAT IIα) in the methionine cycle [57], indicating that HDAC3 can deacetylate a metabolic enzyme and may play an important role in regulating metabolic pathway(s). Supporting this notion, we show in this study that another metabolic enzyme, PGK1, is a direct substrate of HDAC3 and that HDAC3-mediated PGK1 deacetylation plays a signaling role in regulating PGK1 activity/function upon insulin stimulation. Moreover, we demonstrate that the PI3K/AKT/mTOR pathway regulates PGK1 K220 acetylation, in part, via affecting HDAC3 Ser424 phosphorylation, suggesting a potential mechanism for HDAC3 Ser424 phosphorylation regulating PGK1 K220 acetylation. Upon insulin stimulation, the PI3K/AKT/mTOR pathway induces HDAC3 Ser424 phosphorylation, which increases the deacetylase activity of HDAC3 and/or enhances the protein association between HDAC3 and PGK1, leading to PGK1 K220 deacetylation and enzyme activation (Fig 7I). mTOR is a central cell growth controller and is potently activated by insulin [58]. In addition, mTOR is also regulated by nutrient availability and cellular energy status to control cellular metabolism. Our data provides a direct link of mTOR activation to glycolysis, which is achieved by mTOR-mediated HDAC3 phosphorylation and PGK1 deacetylation, thus leading to PGK1 activation. These results also provide an example illustrating how cells integrate different pathways such as extracellular growth signaling and intracellular metabolic flux by a crosstalk involving different type of protein modifications such as phosphorylation and acetylation.

In addition, we have also identified KAT9 as a potential acetyltransferase of PGK1. KAT9/ELP3, which encodes the catalytic subunit of the histone acetyltransferase elongator complex, has previously been identified as an α-tubulin acetyltransferase in mouse neurons [59]. Previously, we reported that KAT9 is the potential acetyltransferase of glucose-6-phosphate dehydrogenase (G6PD), which is a key enzyme in the pentose phosphate pathway and plays an essential role in the oxidative stress response by producing NADPH [60]. In this study, we show that KAT9 as the potential acetyltransferase of PGK1 interacts with and increases PGK1 acetylation, thereby inhibiting PGK1 activity. However, KAT9 may not contribute to cells sensing insulin signal to regulate PGK1 acetylation and function, as neither *KAT9* expression nor KAT9-PGK1 interaction is changed upon insulin treatment.

### Acetylation of PGK1 Coordinates Energy Metabolism and Oxidative Stress Response

Our study also links the regulation of PGK1 activity by acetylation to cellular response to oxidative stress. We show that replacement of endogenous of PGK1 with an acetylation-mimetic K220Q mutant results in a significant decrease in NADPH production and higher susceptibility of cells to oxidative stress. The underlying mechanism for PGK1 controlling NADPH production remains unclear. We propose that a general inhibition of glycolysis may at least in part explain the reduction in NADPH production. In addition, we also show that both 3-PG and serine levels are significantly reduced in cells with *PGK1* knockdown and put-back of the acetylation mimetic K220Q mutant as compared to wild-type rescued cells (Fig 7B). 3-PG is essential for de novo serine biosynthesis [44]. Serine can be converted to glycine by serine hydroxymethyl transferase, a reaction that yields one carbon units, which enter the tetrahydrofolate cycle and are critical for NADPH production [61,62]. Whether PGK1 K220 acetylation inhibits PGK1 catalytic activity, leading to reduced production of 3-PG, disturbed serine biosynthetic flux, and subsequently reduced NADPH production, still needs further investigation.

Recent work has suggested a critical role of serine metabolism in cancer pathogenesis [63–65]. Supporting this notion, *PHGDH* gene expression is up-regulated in diverse cancer cells, such as esophageal adenocarcinoma, triple-negative breast cancer, and melanoma [66]. Moreover, cancer cells with *PHGDH* amplification were found to exhibit increased metabolic flux into serine biosynthesis, which is known to play a dual role in redox balance and providing nucleotide units to support cancer cell proliferation [5,67]. As noted above, K220 acetylation inhibits PGK1 activity/function and reduces the 3-PG and serine levels, implying that modulation of PGK1 activity/function by acetylation may serve as a promising anti-cancer strategy through regulating serine metabolism.

Moreover, the clinical implication of PGK1 dysfunction has been highlighted by chronic haemolysis with progressive neurological impairment in PGK1-deficient patients [68–70]. Two metabolic alterations, a decreased steady-state level of ATP and an increased 2,3-BPG, in red blood cells with human PGK1 deficiency have been proposed to cause hemolytic anemia [71]. It is known that hemolytic anemia also contributes to the inability of erythrocyte cells to produce NADPH and withstand harmful oxidants [72]. Likely, PGK1 dysfunction-associated chronic haemolysis and neurological impairment are in part caused by oxidative stress due to increased levels of oxidative damage and decrease levels of antioxidants, such as reductant NADPH. In this study, we have provided evidence showing that HDAC3-dependent acetylation regulates the function of PGK1 in both glycolytic ATP production and NADPH redox. Therefore, future therapeutic intervention(s) to modulate PGK1 activity via HDAC3-mediated deacetylation may serve as a potential target for treating related diseases, such as hemolytic anemia and brain disorders associated with PGK1 dysregulation.

## Methods and Materials

### Animal Research

All the animal experiments were carried out in accordance with the National Institutes of Health guidelines for the Care and Use of Laboratory Animals and the regulations of Fudan University for animal experimentation. MaleBALB/c mice (6–8 wk old, 20–25 g body weight) were purchased from the Fudan Animal Center. Animals were given unrestricted access to a standard diet and tap water. Blood was collected from tail vein at different time points post intraperitoneal injection of insulin (5 U/kg body weight), and blood glucose levels were determined by using a glucose detection kit (Roche). Before being humanely killed, mice were anesthetized with sodium pentobarbital (25 mg pentobarbital/kg body weight, ip). After that, mouse livers and kidneys were harvested and then homogenized using the Tissuelyser-24 (Shanghai JingXin) in 0.5% NP-40 buffer containing protease inhibitor cocktail, and lysed on ice for 30 min. To determine the acetylation level of endogenous Pgk1, tissue lysates were incubated with the Pgk1 antibody (Santa Cruz) for 1 hr, followed by incubating with Protein-A beads (Upstate) for another 2 hr at 4°C.

### Antibodies

The Rabbit anti-pan-acetyllysine antibody was generated as previously described [73]. To generate acetyl-lysine 220 specific antibody of PGK1, synthetic peptide VADKIQLINNMLDK was coupled to KLH as antigen to immunize rabbit (Shanghai Genomic Inc). For more detail information about the other antibodies used in this study, please refer to the S1 Text.

### PGK1 Enzyme Activity Assay

Flag-tagged PGK1 protein was overexpressed in HEK293T cells, immumoprecipitated and eluted by 250 mg/ml Flag peptides (Gilson Biochemical) dissolved in PBS (pH 7.5). The PGK1 activity assay was carried out as previously described [74]. The reactions were started by adding enzyme into the buffer containing 80 mM pH 7.6 triethanolamine, 8.0 mM MgCl_2_, 0.25 mM NADH, 2.4 mM ATP, 12 mM 3-phosphoglycerate and 50 μg/ml glyceraldehyde-3-phosphate dehydrogenase in a total volume of 0.3 ml, and assayed at 25°C. By monitoring the reduction of NADH fluorescence (Ex350 nm, Em470 nm), the specific PGK1 activity was measured using HITACHI F-4,600 fluorescence spectrophotometer.

### Expression of the K220 Site-Specific Acetylated PGK1 Protein

The K220 site-specific acetylated PGK1 was expressed in *E*. *coli* as previously described [32,33]. In short, the ORF of PGK1 was cloned into pTEV-8 vector with amber codon being incorporated at lysine 220 (AAG to TAG by site-directed mutagenesis). The *E*. *coli* strain BL21 (DE3) was transformed with three plasmids, pAcKRS-3, pCDF PylT-1, and pTEV-8-PGK1. Cells were grown overnight in LB containing spectinomycin (50 μg/ml), kanamycin (50 μg/ml), and ampicillin (150 μg/ml) at 37°C till OD_600_ reached 0.6–0.8. The culture was added with 20 mM NAM and 10 mM N-acetyllysine (Sigma-Aldrich). Protein expression was induced at 37°C by addition of 0.5 mM of isopropyl-1-thio-D-galactopyranoside (IPTG) for 3 hr. Afterward, the cells were harvested, the K220-acetylated PGK1 protein was purified and then stored at −80°C till further analysis.

### Isothermal Calorimetric Titration (ITC) Assay

ITC assay was performed by using a MicroCal VP-ITC type microcalorimeter (MicroCal Inc.). Briefly, temperature equilibration was allowed for 1–2 hr till to 20°C prior to the experiment. PGK1 protein and ADP (Sigma) were dialyzed against 40 mM Tris-HCl (pH 7.4), 50 mM KCl, and 10 mM MgCl_2_. All solutions were thoroughly degassed before being used by centrifugation at 13,000 rpm for 20 min. The experiment was conducted by consecutively injecting 50 μM ADP solution into the calorimetric cell containing 50 μM purified PGK1. The titration enthalpy data was corrected for the small heat changes in control titrations of ADP solution into the dialysis buffer.

### Generation of *PGK1* Knockdown and Put-Back Stable Cell Lines

To generate stable *PGK1* knockdown cell pools in HEK293T cells, shRNA targeting *PGK1* was constructed, and retrovirus was produced using a two-plasmid packaging system as previously described [75]. The shRNA targeting sequence for *PGK1* is 5′-GCTTCTGGGAACAAGGTTAAA-3′. The pMKO.1-puro shRNA construct was co-transfected with vectors expressing the *gag* and *vsvg* genes into HEK293T cells. Retroviral supernatant was harvested 36 hr after transfection, and mixed with 8 μg/mL polybrene to increase the infection efficiency. HEK293T cells were infected with the retrovirus and selected in 1 μg/ml puromycin for 1 wk.

To generate *PGK1* knockdown and put-back stable cell pools in HEK293T cells, two silent nucleotide substitutions were introduced into Flag-tagged human wild-type or K220 mutant PGK1 in the sequence corresponding to the shRNA targeted region. Both shRNA resistant PGK1 were cloned into the retroviral pQCXIH-hygro vector and co-transfected with vectors expressing the gag and vsvg genes in HEK293T cells to produce retrovirus. Retroviral supernatant was harvested 36 hr after transfection, and mixed with 8 μg/mL polybrene to increase the infection efficiency. HEK293T cells with *PGK1* knockdown were infected with the retrovirus and selected in 1 μg/ml puromycin and 2 μg/ml hygromycin for 4 wk.

### Measurement of Intracellular NADPH/NADP^+^ Ratio

The intracellular NADPH/NADP^+^ was measured by enzymatic cycling methods as previously described [76,77]. Briefly, cells were counted and seeded in 10 cm dishes at a density of 1.5 × 10^6^. After 24 hr, cells were lysed in 400 μl of extraction buffer (20 mM NAM, 20 mM NaHCO_3_, 100 mM Na_2_CO_3_) and centrifuged at 1,200 g for 15 min. 150 μl of the supernatant was incubated in a heating block for 30 min at 60°C for NADPH extraction. And then, 20 μl of the cell extract with 160 μl of NADP-cycling buffer (100 mM Tris-HCl, pH8.0; 0.5 mM thiazolylblue; 2 mM phenazine ethosulfate; 5 mM EDTA) containing 1.3 U of G6PD was added to a 96-well plate. After incubation for 1 min at 30°C in darkness, 20 μl of 10 mM G6P was added to well, and measured the change of absorbance at 570 nm every 30 s for 10 min at 30°C by using SpectraMax M5 Microplate Reader (Molecular Devices). Subtracting NADPH (heated sample) from the total of NADP^+^ and NADPH (unheated sample) was the level of NADP^+^.

### Statistical Analysis

Statistical analyses were performed with a two-tailed unpaired Student's *t* test. Almost all data shown represent the results obtained from triplicated independent experiments with standard deviation of the mean (mean ± S.D.). The values of *p* < 0.05 were considered statistically significant. The numerical data and statistical analysis used in all figures are included in S1 Data.

## Supporting Information

S1 Data Excel spreadsheet containing, in separate sheets, the underlying numerical data and statistical analysis for Figs 1A, 1B, 1C, 1E, 1F, 2A, 2D, 3C, 3D, 3E, 3G, 4A, 4B, 4D, 7A, 7B, 7C, 7D, 7E, 7F, 7G, 7H, S3, S5, S6, S7A, S8, S9, S13, S14B, S14C, S15A and S15B. (XLSX)

S1 Fig PGK1 acetylation is increased by treatment with TSA, but not NAM. Flag-PGK1 was overexpressed in HEK293T cells followed by treatment with nicotinamide (NAM, 10 mM) (**A**) and trichostatin (TSA, 5 μM) (**B**) as indicated. After purification by immunoprecipitation, the acetylation level of ectopically expressed Flag-PGK1 was analyzed by western blot using a pan-anti-acetyllysine antibody (α-Pan-Ac) and normalized against Flag.
(TIF)

S2 Fig K220 of PGK1 is evolutionarily conserved from yeast to human. The sequence alignment of PGK1 from different species was compared, and the putative acetylated lysine residues of PGK1 identified by MS study were colored in red.
(TIF)

S3 Fig K220R mutant displays a negligible response in changing acetylation and enzyme activity upon NAM+TSA treatment. Flag-tagged wild-type PGK1, K75R or K220R mutant were each overexpressed in HEK293T cells, following treatments with or without NAM (10 mM for 4 hr) and TSA (5 μM for 12 hr). Acetylation levels and activity of PGK1 were determined by western blot and enzymatic activity assay, respectively. Relative PGK1 acetylation levels were normalized against Flag. Note that the acetylation levels of wild-type, K75R, and K220R PGK1 in cells treated without or with NAM + TSA (i.e. the α-Pan-Ac bands) were determined on the same gel, while the α-Flag loading control samples were loaded to two separate gels. Shown are average values with standard deviation (S.D.) of triplicated experiments. ** denotes *p* < 0.01 for the indicated comparison; n.s. = not significant. The numerical data and statistical analysis used in the figures are included in S1 Data.
(TIF)

S4 Fig Characterization of the specificity of the α-acetyl-PGK1 (K220) antibody. **(**A) Different amounts of acetyl-K220 peptide or unmodified peptide were spotted on nitrocellulose membrane, and the antibody specificity was determined by dot-blot assay. (**B**) HEK293T cell lysates were separately used for western blot with the α-PGK1 antibody (lane 1), α-acetyl-PGK1 (K220) antibody (lane 2), the α-acetyl-PGK1 (K220) antibody incubated with acetylated peptide (lane 3), or unmodified peptide (lane 4). The image is comprised from four separate western blots. (**C**) Flag-tagged vector, wild-type, or K220Q/R mutant PGK1 was transfected into HEK293T cells and the acetylation level of each purified protein was measured by western blot using the site-specific α-acetyl-PGK1 (K220) antibody or pre-immune serum.
(TIF)

S5 Fig The knockdown efficiency of a siRNA library with three siRNAs targeting each of the indicated 19 *KAT* genes. The siRNA oligonucleotide was transiently transfected into HEK293T cells, and the mRNA expression of each *KAT* gene was determined by quantitative real-time PCR at 48 hr post transfection. Shown are average values with standard deviation (S.D.) of triplicated experiments. The numerical data and statistical analysis used in the figures are included in S1 Data.
(TIF)

S6 Fig A dynamic change of Pgk1 K220 acetylation in mouse kidney after insulin injection. Insulin (5 U/kg body weight) was intraperitoneally injected into BALB/c wild-type mice (*n* = 3 per group). At the indicated time points post injection, mouse kidney samples were harvested, and the K220 acetylation levels of endogenous Pgk1 were examined by western blot. The relative Pgk1 K220 acetylation levels were normalized against endogenous Pgk1 protein. The numerical data and statistical analysis used in the figures are included in S1 Data.
(TIF)

S7 Fig Neither *KAT9* mRNA expression nor KAT9-PGK1 association is changed after insulin treatment. (**A**) HEK293T cells were treated with insulin at the indicated concentrations for 2 hr, and *KAT9* mRNA expression was determined by quantitative real-time PCR. The expression of *KAT9* was normalized against β-actin. Shown are average values with standard deviation (S.D.) of triplicated experiments. n.s. = not significant. (**B**) Flag-tagged PGK1 and/or HA-tagged KAT9 were transiently overexpressed in HEK293T cells, and the cells were then treated without or with insulin (50 nM, 2 hr). PGK1 protein was purified by IP with Flag beads, following western blot to detect its K220 acetylation level and interaction with KAT9. The numerical data and statistical analysis used in the figures are included in S1 Data.
(TIF)

S8 Fig *HDAC3* mRNA expression is not changed after insulin treatment. HEK293T cells were treated with insulin at the indicated concentrations for 2 hr, and *HDAC3* mRNA expression was determined by quantitative real-time PCR. The expression of *HDAC3* was normalized against β-actin. Shown are average values with standard deviation (S.D.) of triplicated experiments. n.s. = not significant. The numerical data and statistical analysis used in the figures are included in S1 Data.
(TIF)

S9 Fig HDAC3 S424A mutant exhibits reduced deacetylase activity and impaired protein association with PGK1. HA-tagged proteins of wild-type and HDAC3 S424A mutant were transiently overexpressed in HEK293T cells. Cell extracts were immunoprecipitated and assayed for the histone deacetylase activity of HDAC3 as described in S1 Text. Moreover, the level of HDAC3 Ser424 phosphorylation and the protein association between ectopically expressed HDAC3 and endogenous PGK1 were determined by western blot. Shown are average values with standard deviation (S.D.) of triplicated experiments. The numerical data and statistical analysis used in the figures are included in S1 Data.
(TIF)

S10 Fig The PI3K/AKT pathway regulates PGK1 K220 acetylation. (**A, B**) Inhibition of PI3K increases the K220 acetylation level of ectopically expressed PGK1 and impairs the protein interaction between ectopic proteins of PGK1 and HDAC3. Flag-tagged PGK1 and HA-tagged HDAC3 were transiently co-overexpressed in HEK293T cells, and then these transfected cells were treated with two PI3K inhibitors, LY294002 (**A**) or Wortmannin (**B**), at the indicated concentrations for 4 hr and 2 hr, respectively. PGK1 proteins were purified by Flag beads, following western blot to the detect K220 level acetylation of Flag-PGK1 and its interacting HA-HDAC3.
(TIF)

S11 Fig EGF cannot potently activate mTOR to change HDAC3 S424 phosphorylation and PGK1 K220 acetylation. Flag-tagged PGK1 was ectopically expressed in HEK293T cells treated with EGF (50 μg/L) for the indicated time periods. Endogenous proteins of ERK1/2, S6K, HDAC3 and their phosphorylation were detected by western blotting. PGK1 proteins were purified by Flag beads, and PGK1 K220 acetylation was determined by western blot.
(TIF)

S12 Fig Generation of *PGK1* knockdown and put-back stable cells. Stable HEK293T cells with knockdown of endogenous *PGK1* were transfected with retroviral plasmids expressing the indicated proteins. *PGK1* knockdown efficiency and re-expression were determined by western blot.
(TIF)

S13 Fig Determination of cell apoptosis in *PGK1* knockdown and put-back stable cells subjected to rotenone treatment. Stable HEK293T cells were treated with rotenone (100 nM) for 6 hr, and then the percentage of viable cells was determined by flow cytometric analysis using Annexin V and propidium iodode (PI) antibodies as described in S1 Text. Shown are average values with standard deviation (S.D.) of triplicated experiments. The numerical data and statistical analysis used in the figures are included in S1 Data.
(TIF)

S14 Fig Determination of the glycolytic function in *PGK1* knockdown and put-back stable cells. Extracellular acidification rate (ECAR) was determined in HEK293T cells with *PGK1* knocking-down and putting-back using a Seahorse Bioscience XFe 96 analyzer system as described in S1 Text (as shown in **A**). The glycolytic function of rescued cells expressing wild-type PGK1 (blue circle), K220Q mutant PGK1 (red circle), and K220R mutant PGK1 (green circle) was compared (**B**). The parameters reflecting glycolysis, glycolytic capacity, and glycolytic reserve were determined by calculating Rate Measurement Equation (**C**). Shown are average values with standard error of the mean (S.E.M.) of triplicated experiments. The numerical data and statistical analysis used in the figures are included in S1 Data.
(TIF)

S15 Fig Determination of glucose consumption and glycogen content in *PGK1* knockdown and put-back stable cells. Relative glucose consumption (**A**) and glycogen storage levels (**B**) in the indicated stable HEK293T cells were measured as described in S1 Text. Shown are average values with standard deviation (S.D.) of triplicated experiments. ** denotes *p* < 0.01 for the indicated comparisons. The numerical data and statistical analysis used in the figures are included in S1 Data.
(TIF)

S16 Fig Determination of ROS-related signaling pathways in *PGK1* knockdown and put-back stable cells subjected to menadione treatment. (**A, B**) Stable HEK293T cells were treated with menadione for the indicated concentrations and periods. The protein expression of PARP and p38 MAPK and their phosphorylation were determined by western blot analysis.
(TIF)

S17 Fig Morphological changes of stable *PGK1* knockdown and put-back cells subjected to menadione treatment. Stable HEK293T cells were treated with menadione of the indicated concentration for 3 hr. The changes in cell morphology were monitored under the microscope. Scale bars are 200 μm.
(TIF)

S1 Table The list of oligonucleotides and primer sequences used in this study. (DOCX)

S1 Text Supplementary materials and methods. For more details about antibodies, plasmids, cell culture and treatment, immunoprecipitation and western blotting, RNA isolation and quantitative real-time PCR, animal experiments, RNA interference, protein concentration determination, protein expression and purification, pull-down assay, in vitro deacetylation assay, HDAC3 activity assay, measurement of intracellular ATP levels, flow cytometry, liquid chromatography mass spectrometry (LC-MS), extracellular acidification rate (ECAR), glucose depletion assay, glycogen content measurement, measurement of intracellular ROS levels, or cell survival assay, please refer to S1 Text.
(DOCX)

## Abbreviations

1, 3-BPG: 1, 3-bisphosphoglycerate
3-PG: 3-phosphoglycerate
A: alanine
ALF: tetrafluoroaluminate
ECAR: extracellular acidification rate
G6PD: glucose-6-phosphate dehydrogenase
GAPDH: glyceraldehyde-3-phosphate dehydrogenase
GCMa: glial cell missing
HA: human influenza hemagglutinin
HDAC3: histone deacetylase 3
IB: immunoblotting
IP: immunoprecipitation
ITC: isothermal titration calorimetry
K220: lysine 220
KATs: lysine acetyltransferases
KDACs: lysine deacetylases
LC-MS: Liquid chromatography-mass spectrometry
MAT IIa: methionine adenosyltransferase Iiα
Mef2: myocyte enhancer factor-2
NAM: nicotinamide
PARP: poly ADP-ribose polymerase
PGK1: phosphoglycerate kinase
PHGDH: phosphoglycerate dehydrogenase
PI3K/AKT/mTOR: phosphoinositide-3-kinase/AKT/mammalian target of rapamycin
S: serine
SRY: sex-determining region Y
TSA: trichostatin A
WT: Wild-type
