# Correction: Insulin and mTOR Pathway Regulate HDAC3-Mediated Deacetylation and Activation of PGK1

**DOI:** 10.1371/journal.pbio.1002287

**Published:** 2015-11-05

**Authors:** Shiwen Wang, Bowen Jiang, Tengfei Zhang, Lixia Liu, Yi Wang, Yiping Wang, Xiufei Chen, Huaipeng Lin, Lisha Zhou, Yukun Xia, Leilei Chen, Chen Yang, Yue Xiong, Dan Ye, Kun-Liang Guan

Fig 2B contains an extra lane in the right end of the top row. The authors have provided a corrected version here.

**Fig 2:**
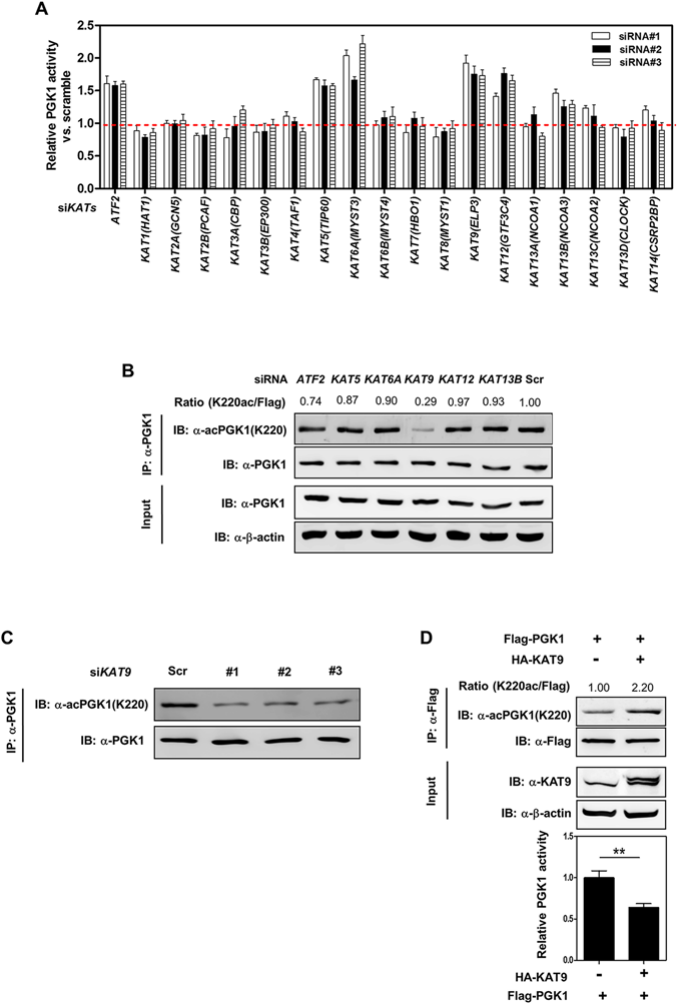
KAT9 acetylates PGK1 and inhibits PGK1 activity. (A) Knockdown of *ATF2*, *KAT5*, *KAT6A*, *KAT9*, *KAT12*, or *KAT13B* leads to increased enzyme activity of endogenous PGK1. HEK293T cells were transiently transfected with the indicated siRNAs. The enzyme activity of endogenous PGK1 was determined as described in “Methods and Materials.” (B, C) *KAT9* knockdown leads to decreased K220 acetylation of endogenous PGK1. HEK293T cells were transiently transfected with the indicated siRNAs. The protein levels and K220 acetylation of endogenous PGK1 were determined by western blot (B). Moreover, HEK293T cells were transiently transfected with three different siRNAs targeting *KAT9*, and the K220 acetylation level of endogenous PGK1 was determined by western blot (C). “Scr” means scramble. Relative PGK1 K220 acetylation levels were normalized against endogenous PGK1. (D) KAT9 overexpression increases PGK1 K220 acetylation and inhibits PGK1 enzyme activity. Flag-tagged PGK1 and HA-tagged KAT9 were transiently co-overexpressed in HEK293T cells, and PGK1 protein was purified by IP with Flag beads, the K220 acetylation level and enzyme activity of PGK1 were determined by western blot and enzyme assay, respectively. Relative PGK1 acetylation levels were normalized against Flag. Shown are average values with standard deviation (S.D.) of triplicated experiments. ** denotes the *p* < 0.01 for the indicated comparison; n.s. = not significant. The numerical data and statistical analysis used in the figures are included in S1 Data. Supporting information can be found in S5 Fig and S1 Table.

## References

[1] Wang S, Jiang B, Zhang T, Liu L, Wang Y, Wang Y, et al (2015) Insulin and mTOR Pathway Regulate HDAC3-Mediated Deacetylation and Activation of PGK1. PLoS Biol 13(9): e1002243 doi:10.1371/journal.pbio.1002243 26356530 10.1371/journal.pbio.1002243 PMC4565669

