# Supplementary figures and images for: Insulin and mTOR Pathway Regulate HDAC3-Mediated Deacetylation and Activation of PGK1
Source: PLoS Biol. 2015 Sep 10;13(9):e1002243. doi: 10.1371/journal.pbio.1002243 (PMC4565669; doi:10.1371/journal.pbio.1002243)

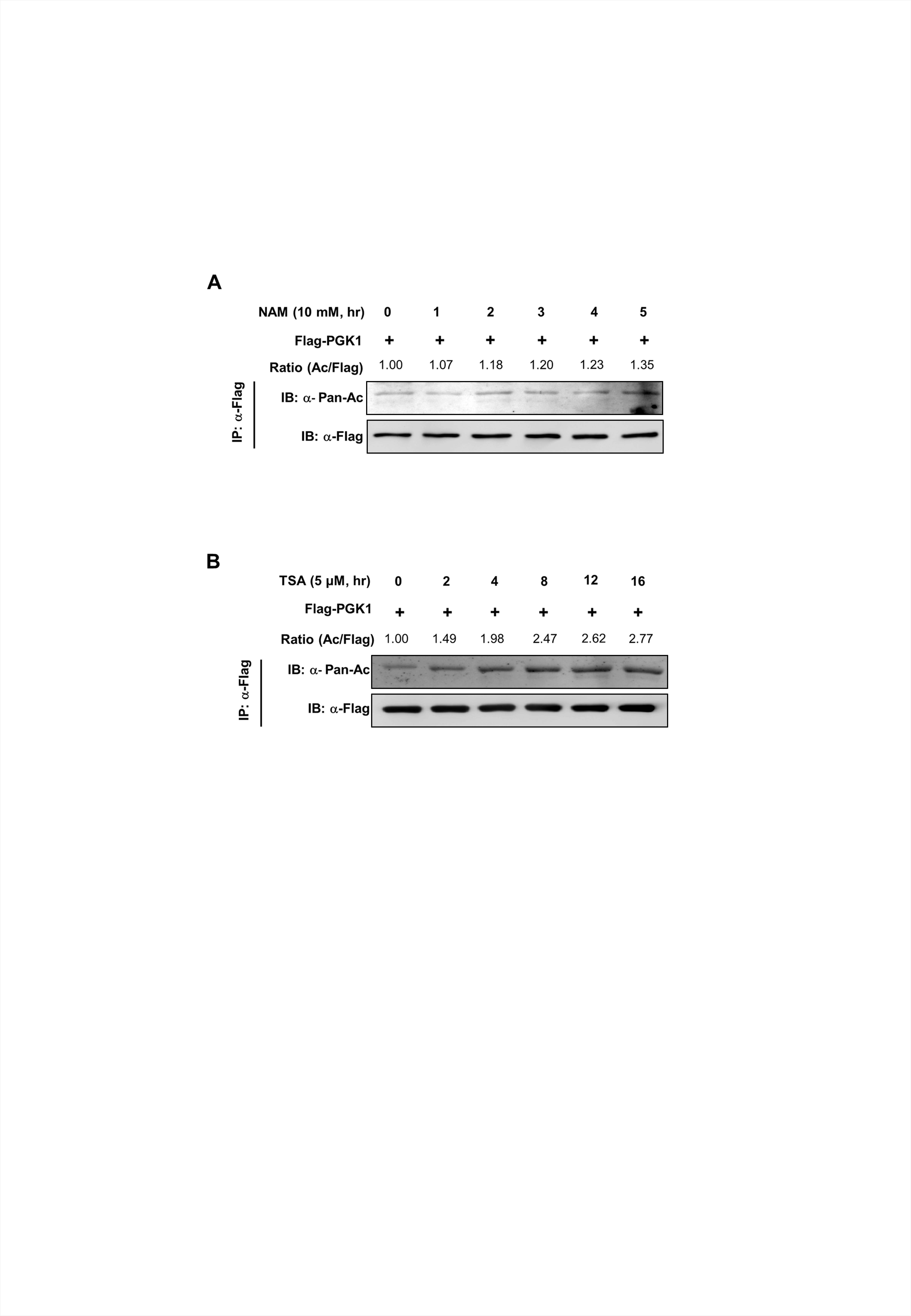

Supplement: S1 Fig — Flag-PGK1 was overexpressed in HEK293T cells followed by treatment with nicotinamide (NAM, 10 mM) (A) and trichostatin (TSA, 5 μM) (B) as indicated. After purification by immunoprecipitation, the acetylation level of ectopically expressed Flag-PGK1 was analyzed by western blot using a pan-anti-acetyllysine antibody (α-Pan-Ac) and normalized against Flag. (TIF) [file pbio.1002243.s002.tif]

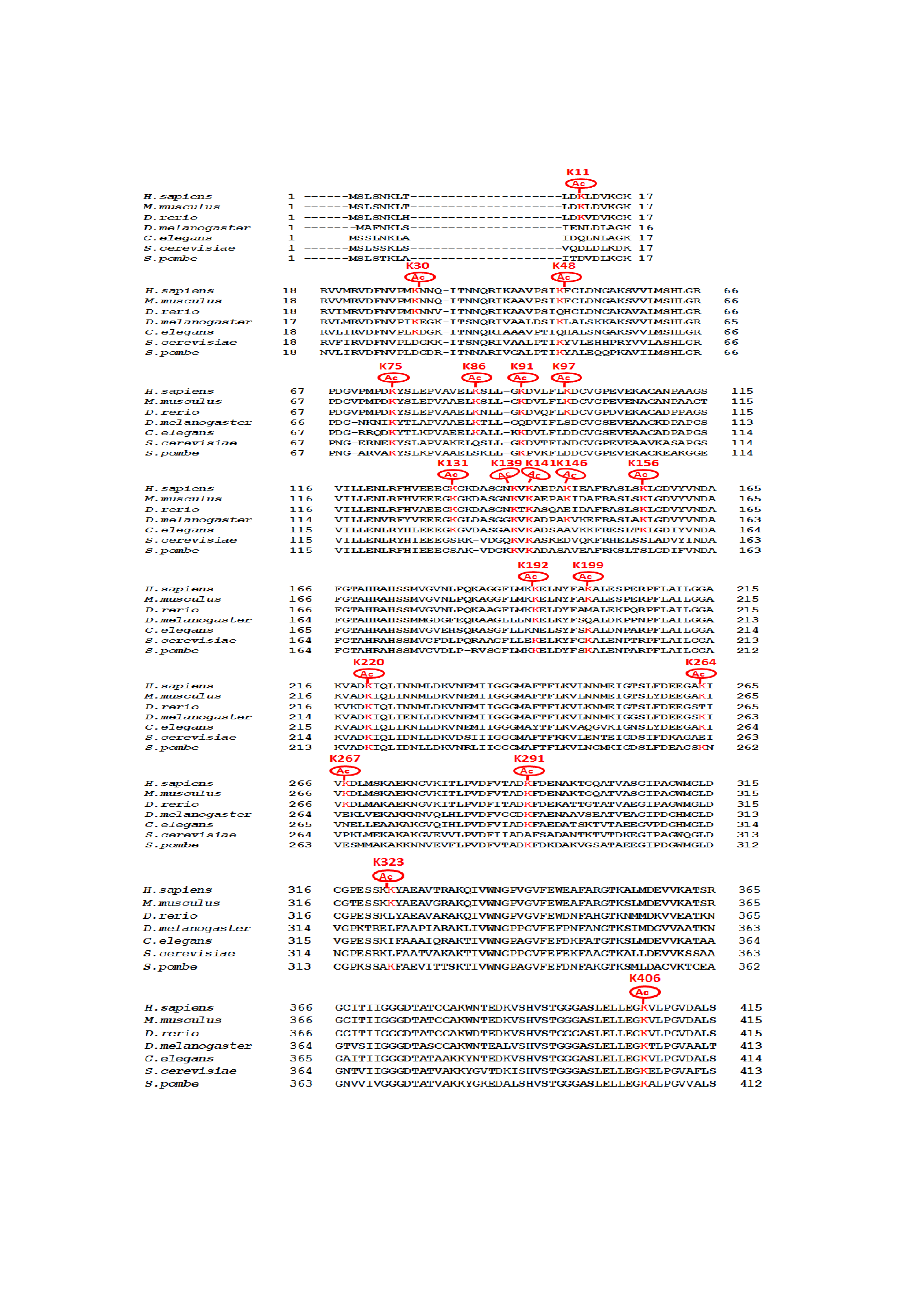

Supplement: S2 Fig — The sequence alignment of PGK1 from different species was compared, and the putative acetylated lysine residues of PGK1 identified by MS study were colored in red. (TIF) [file pbio.1002243.s003.tif]

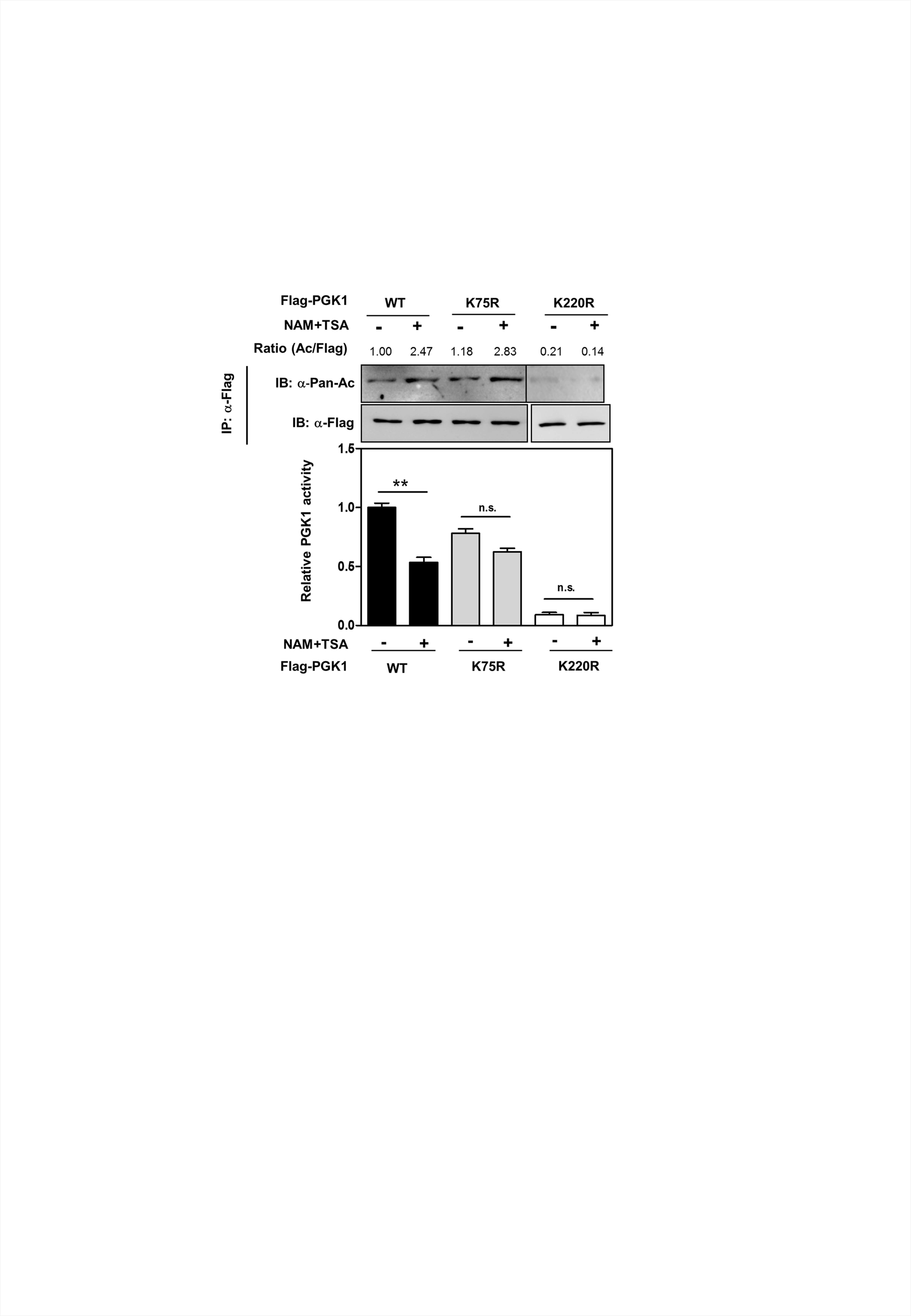

Supplement: S3 Fig — Flag-tagged wild-type PGK1, K75R or K220R mutant were each overexpressed in HEK293T cells, following treatments with or without NAM (10 mM for 4 hr) and TSA (5 μM for 12 hr). Acetylation levels and activity of PGK1 were determined by western blot and enzymatic activity assay, respectively. Relative PGK1 acetylation levels were normalized against Flag. Note that the acetylation levels of wild-type, K75R, and K220R PGK1 in cells treated without or with NAM + TSA (i.e. the α-Pan-Ac bands) were determined on the same gel, while the α-Flag loading control samples were loaded to two separate gels. Shown are average values with standard deviation (S.D.) of triplicated experiments. ** denotes p < 0.01 for the indicated comparison; n.s. = not significant. The numerical data and statistical analysis used in the figures are included in S1 Data. (TIF) [file pbio.1002243.s004.tif]

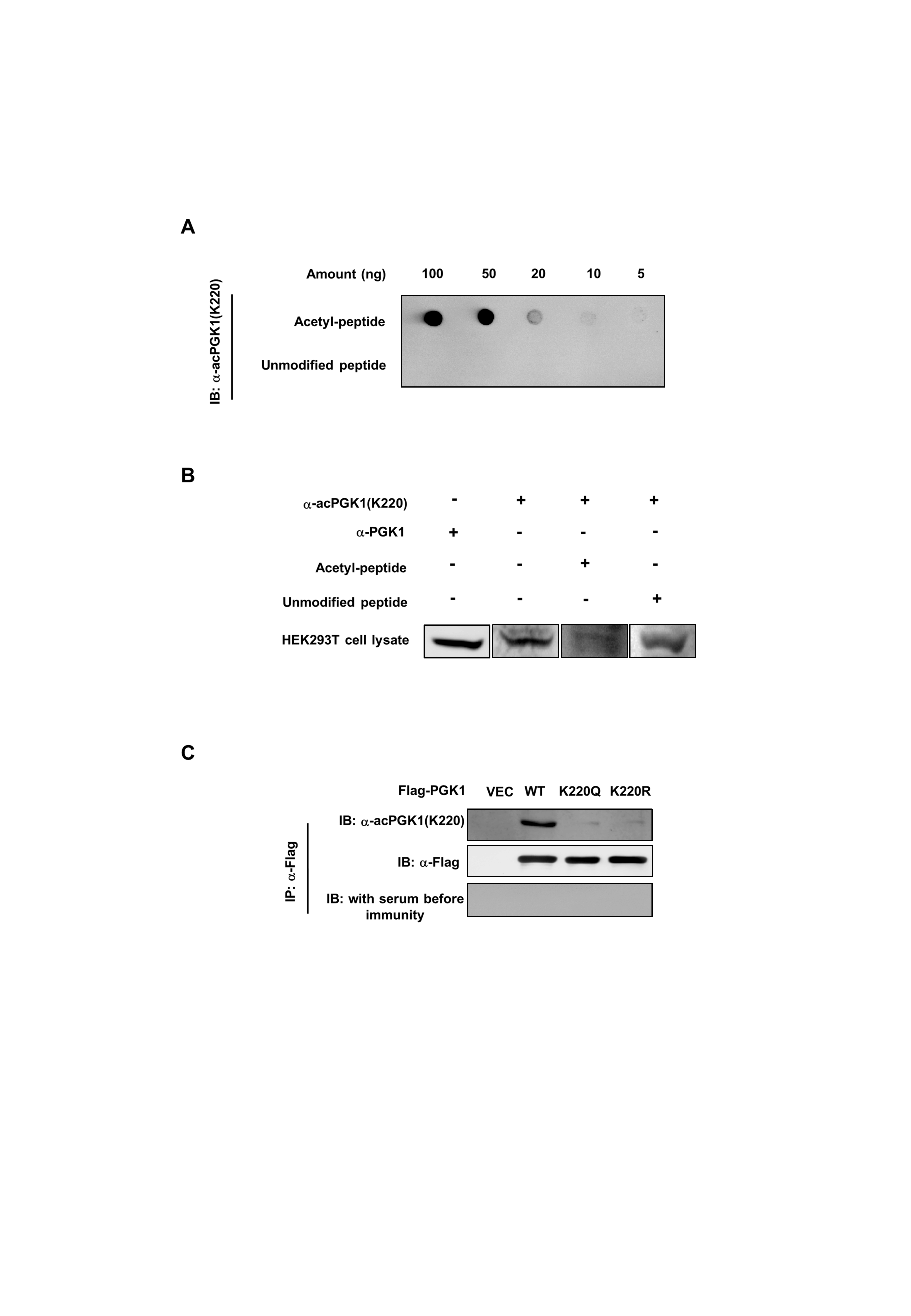

Supplement: S4 Fig — (A) Different amounts of acetyl-K220 peptide or unmodified peptide were spotted on nitrocellulose membrane, and the antibody specificity was determined by dot-blot assay. (B) HEK293T cell lysates were separately used for western blot with the α-PGK1 antibody (lane 1), α-acetyl-PGK1 (K220) antibody (lane 2), the α-acetyl-PGK1 (K220) antibody incubated with acetylated peptide (lane 3), or unmodified peptide (lane 4). The image is comprised from four separate western blots. (C) Flag-tagged vector, wild-type, or K220Q/R mutant PGK1 was transfected into HEK293T cells and the acetylation level of each purified protein was measured by western blot using the site-specific α-acetyl-PGK1 (K220) antibody or pre-immune serum. (TIF) [file pbio.1002243.s005.tif]

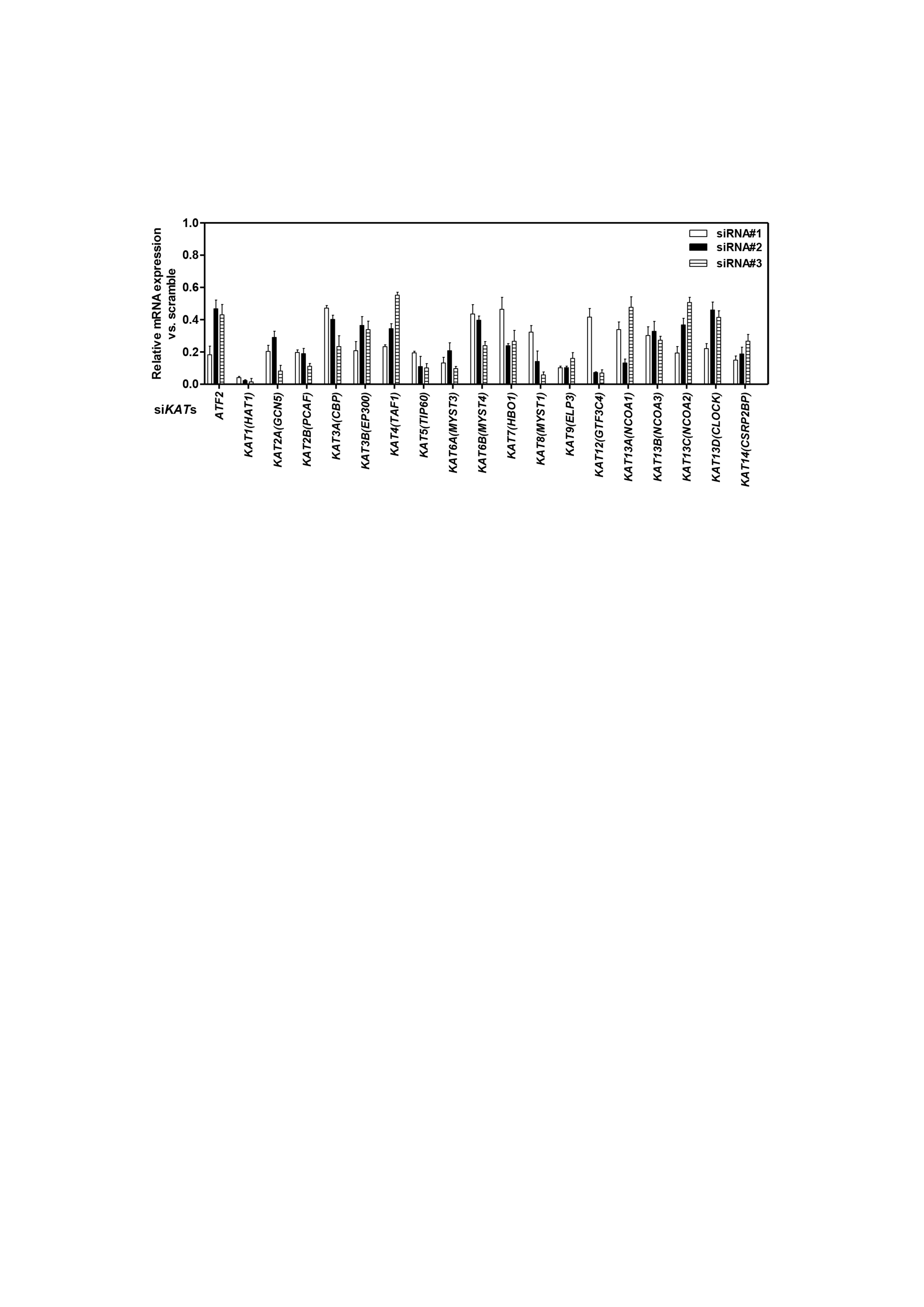

Supplement: S5 Fig — The siRNA oligonucleotide was transiently transfected into HEK293T cells, and the mRNA expression of each KAT gene was determined by quantitative real-time PCR at 48 hr post transfection. Shown are average values with standard deviation (S.D.) of triplicated experiments. The numerical data and statistical analysis used in the figures are included in S1 Data. (TIF) [file pbio.1002243.s006.tif]

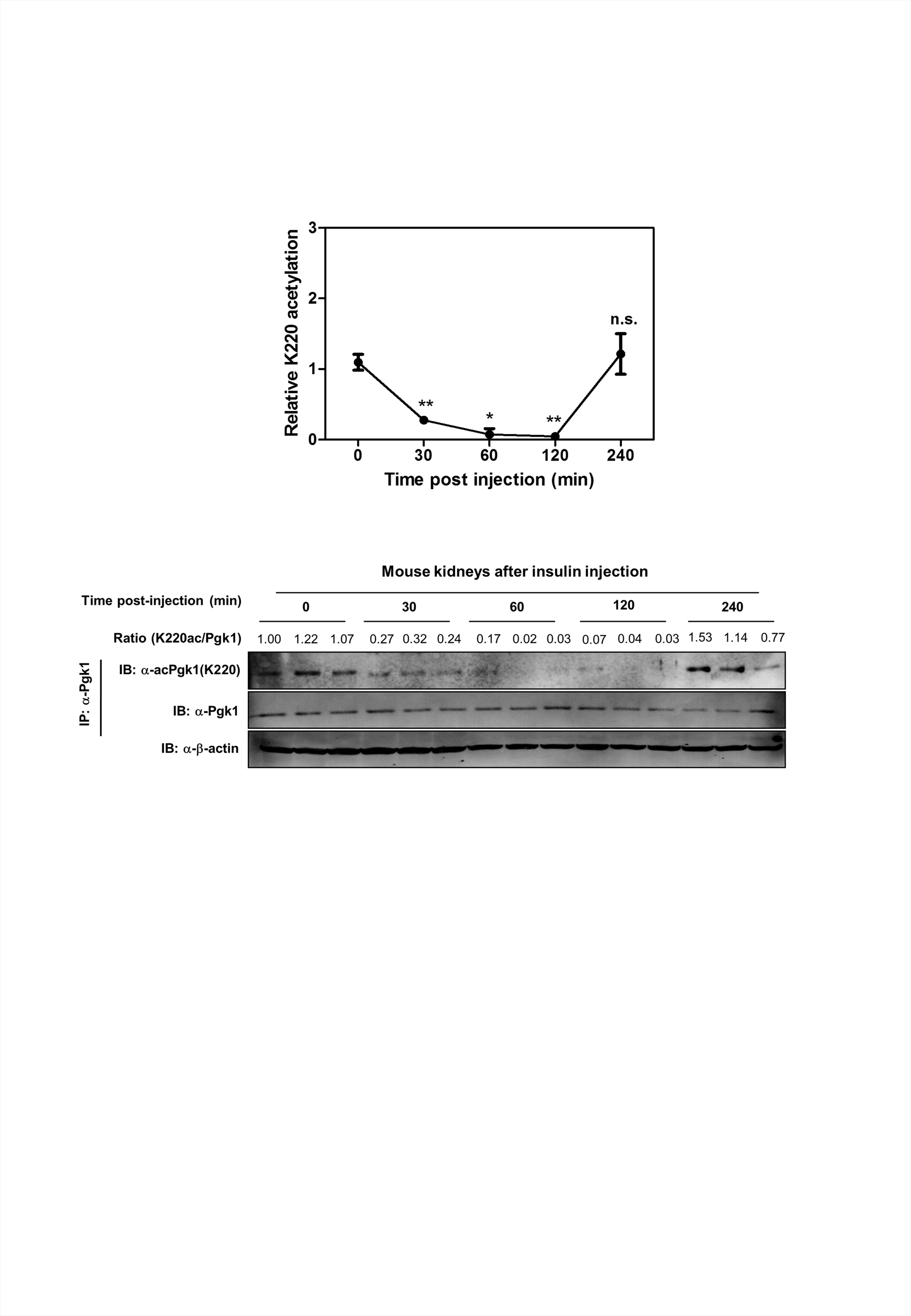

Supplement: S6 Fig — Insulin (5 U/kg body weight) was intraperitoneally injected into BALB/c wild-type mice (n = 3 per group). At the indicated time points post injection, mouse kidney samples were harvested, and the K220 acetylation levels of endogenous Pgk1 were examined by western blot. The relative Pgk1 K220 acetylation levels were normalized against endogenous Pgk1 protein. The numerical data and statistical analysis used in the figures are included in S1 Data. (TIF) [file pbio.1002243.s007.tif]

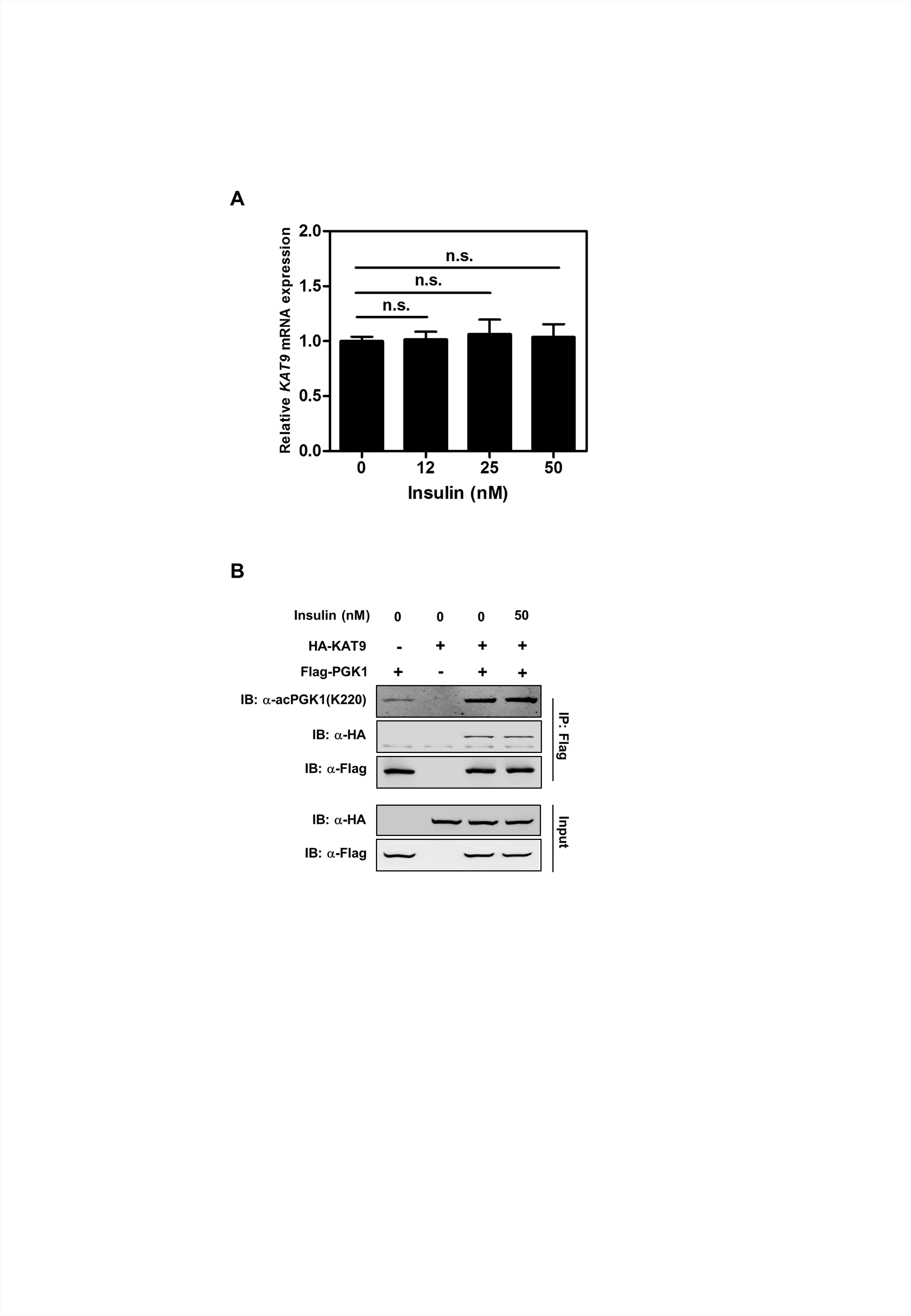

Supplement: S7 Fig — (A) HEK293T cells were treated with insulin at the indicated concentrations for 2 hr, and KAT9 mRNA expression was determined by quantitative real-time PCR. The expression of KAT9 was normalized against β-actin. Shown are average values with standard deviation (S.D.) of triplicated experiments. n.s. = not significant. (B) Flag-tagged PGK1 and/or HA-tagged KAT9 were transiently overexpressed in HEK293T cells, and the cells were then treated without or with insulin (50 nM, 2 hr). PGK1 protein was purified by IP with Flag beads, following western blot to detect its K220 acetylation level and interaction with KAT9. The numerical data and statistical analysis used in the figures are included in S1 Data. (TIF) [file pbio.1002243.s008.tif]

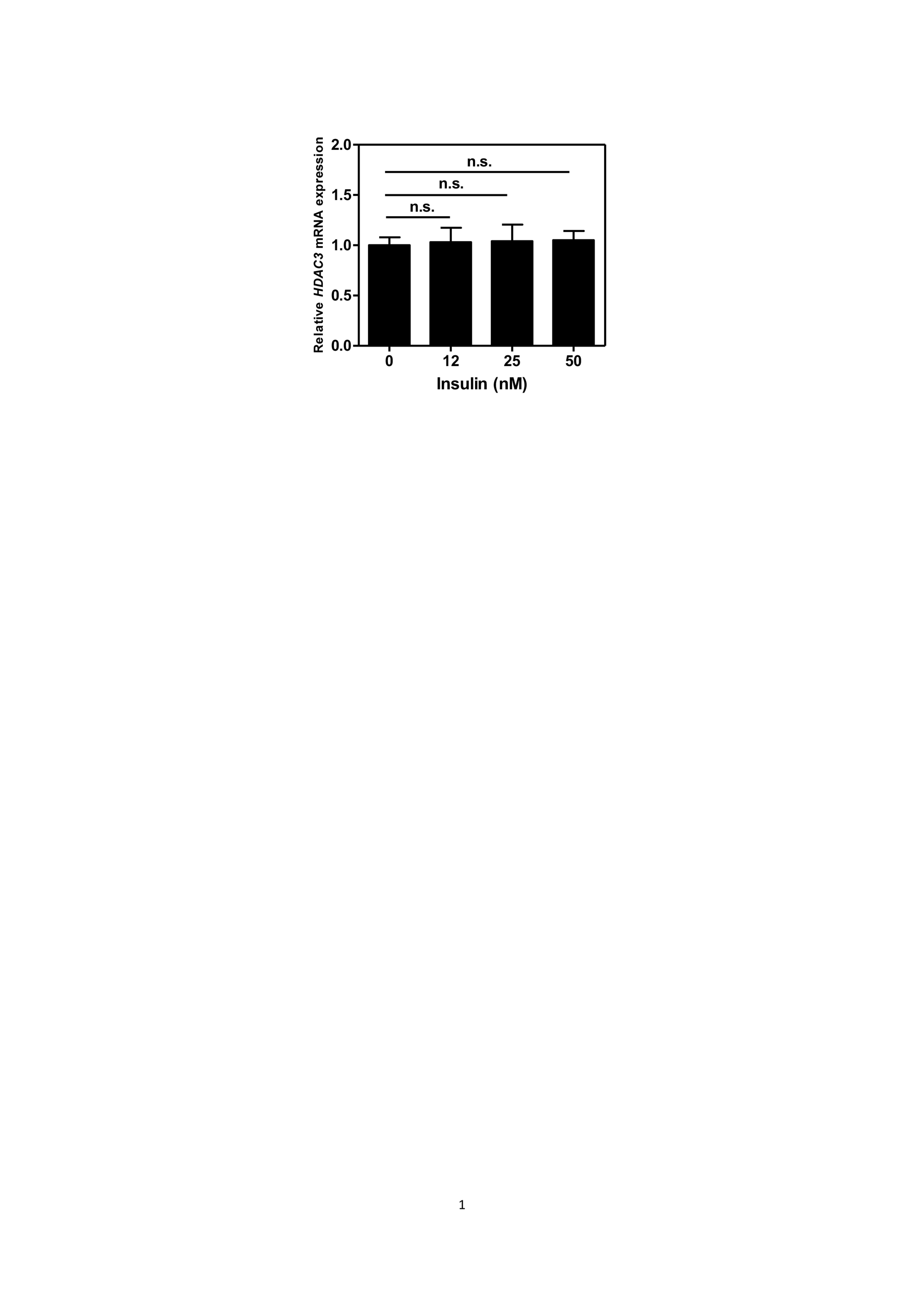

Supplement: S8 Fig — HEK293T cells were treated with insulin at the indicated concentrations for 2 hr, and HDAC3 mRNA expression was determined by quantitative real-time PCR. The expression of HDAC3 was normalized against β-actin. Shown are average values with standard deviation (S.D.) of triplicated experiments. n.s. = not significant. The numerical data and statistical analysis used in the figures are included in S1 Data. (TIF) [file pbio.1002243.s009.tif]

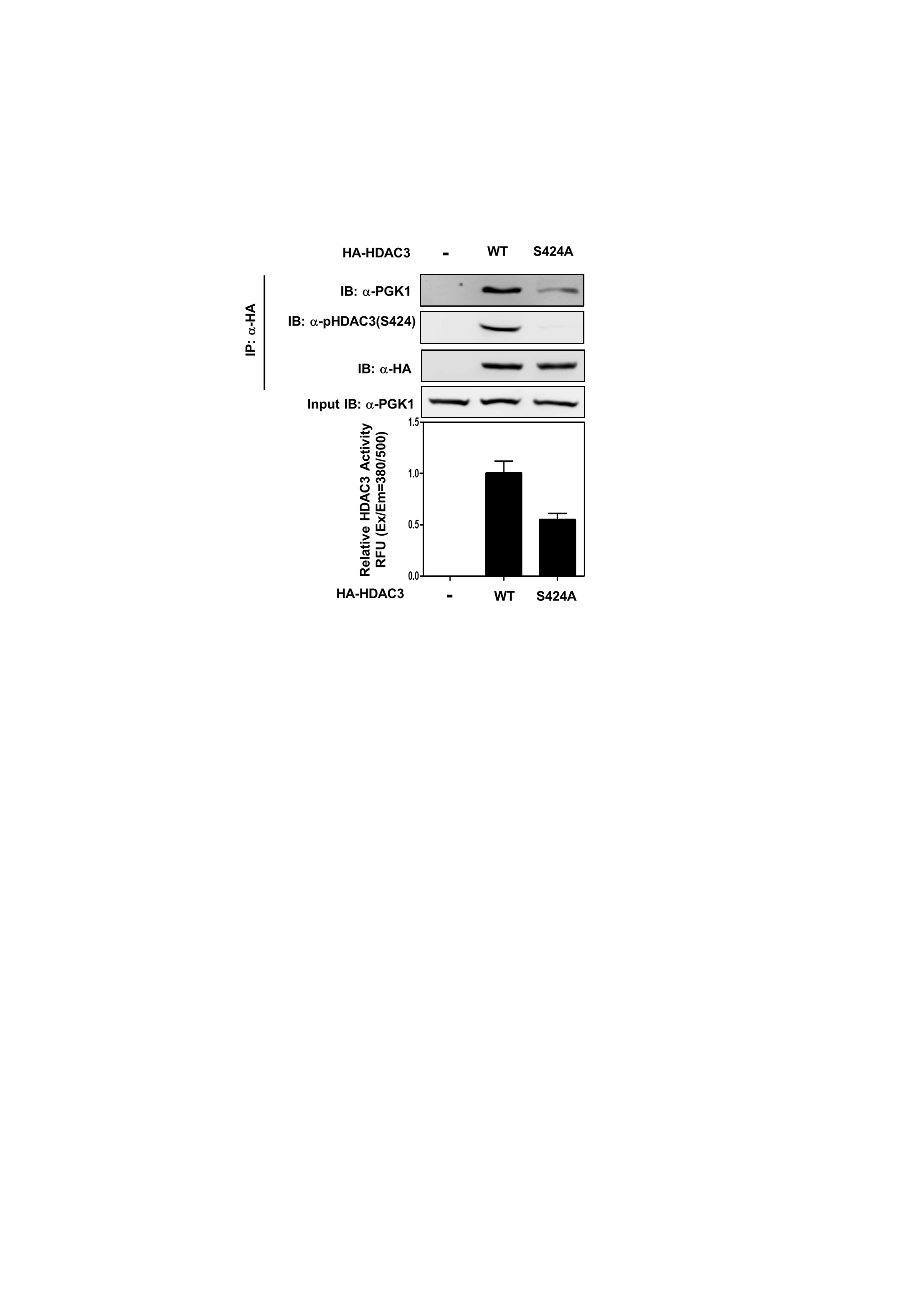

Supplement: S9 Fig — HA-tagged proteins of wild-type and HDAC3 S424A mutant were transiently overexpressed in HEK293T cells. Cell extracts were immunoprecipitated and assayed for the histone deacetylase activity of HDAC3 as described in S1 Text. Moreover, the level of HDAC3 Ser424 phosphorylation and the protein association between ectopically expressed HDAC3 and endogenous PGK1 were determined by western blot. Shown are average values with standard deviation (S.D.) of triplicated experiments. The numerical data and statistical analysis used in the figures are included in S1 Data. (TIF) [file pbio.1002243.s010.tif]

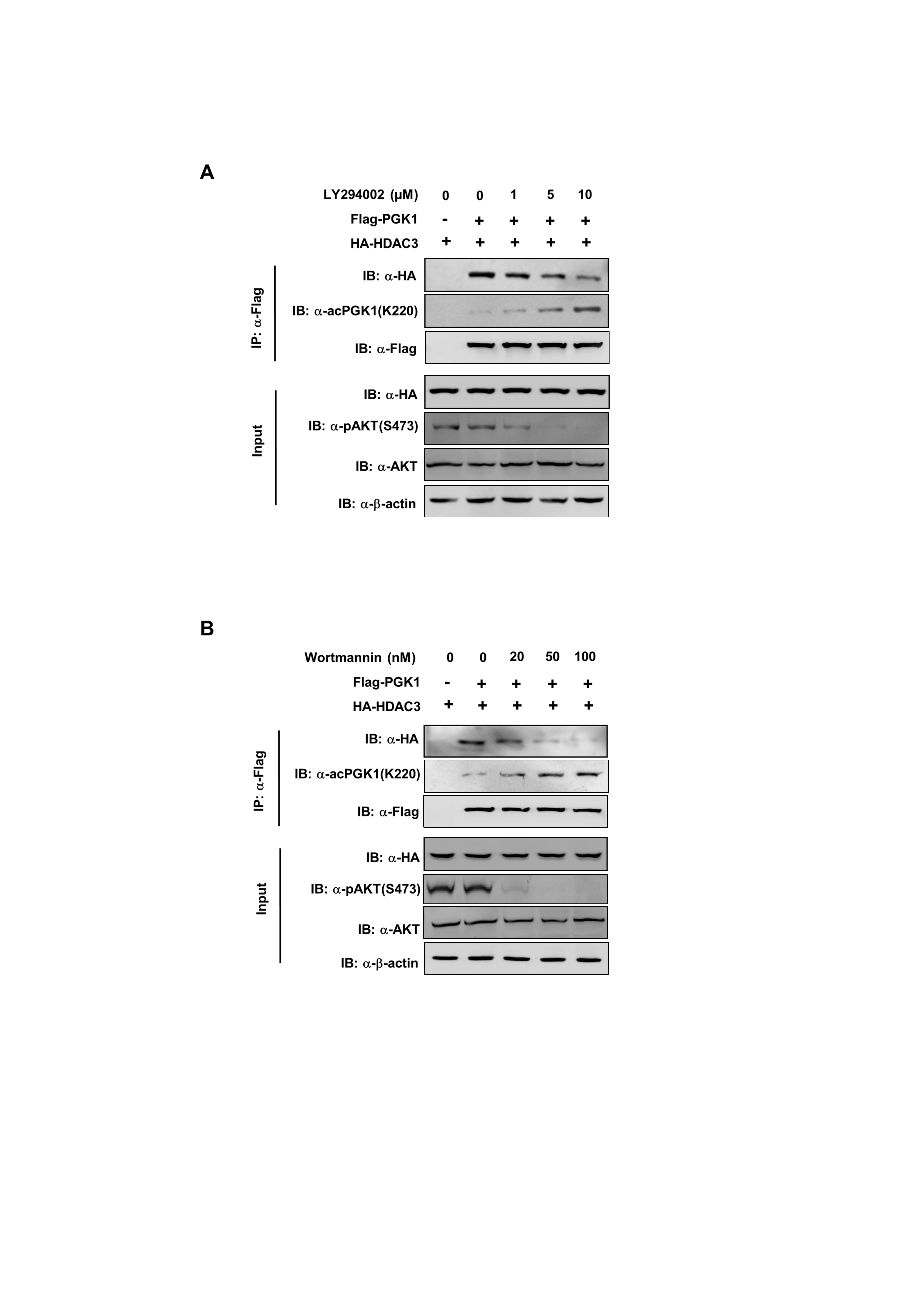

Supplement: S10 Fig — (A, B) Inhibition of PI3K increases the K220 acetylation level of ectopically expressed PGK1 and impairs the protein interaction between ectopic proteins of PGK1 and HDAC3. Flag-tagged PGK1 and HA-tagged HDAC3 were transiently co-overexpressed in HEK293T cells, and then these transfected cells were treated with two PI3K inhibitors, LY294002 (A) or Wortmannin (B), at the indicated concentrations for 4 hr and 2 hr, respectively. PGK1 proteins were purified by Flag beads, following western blot to the detect K220 level acetylation of Flag-PGK1 and its interacting HA-HDAC3. (TIF) [file pbio.1002243.s011.tif]

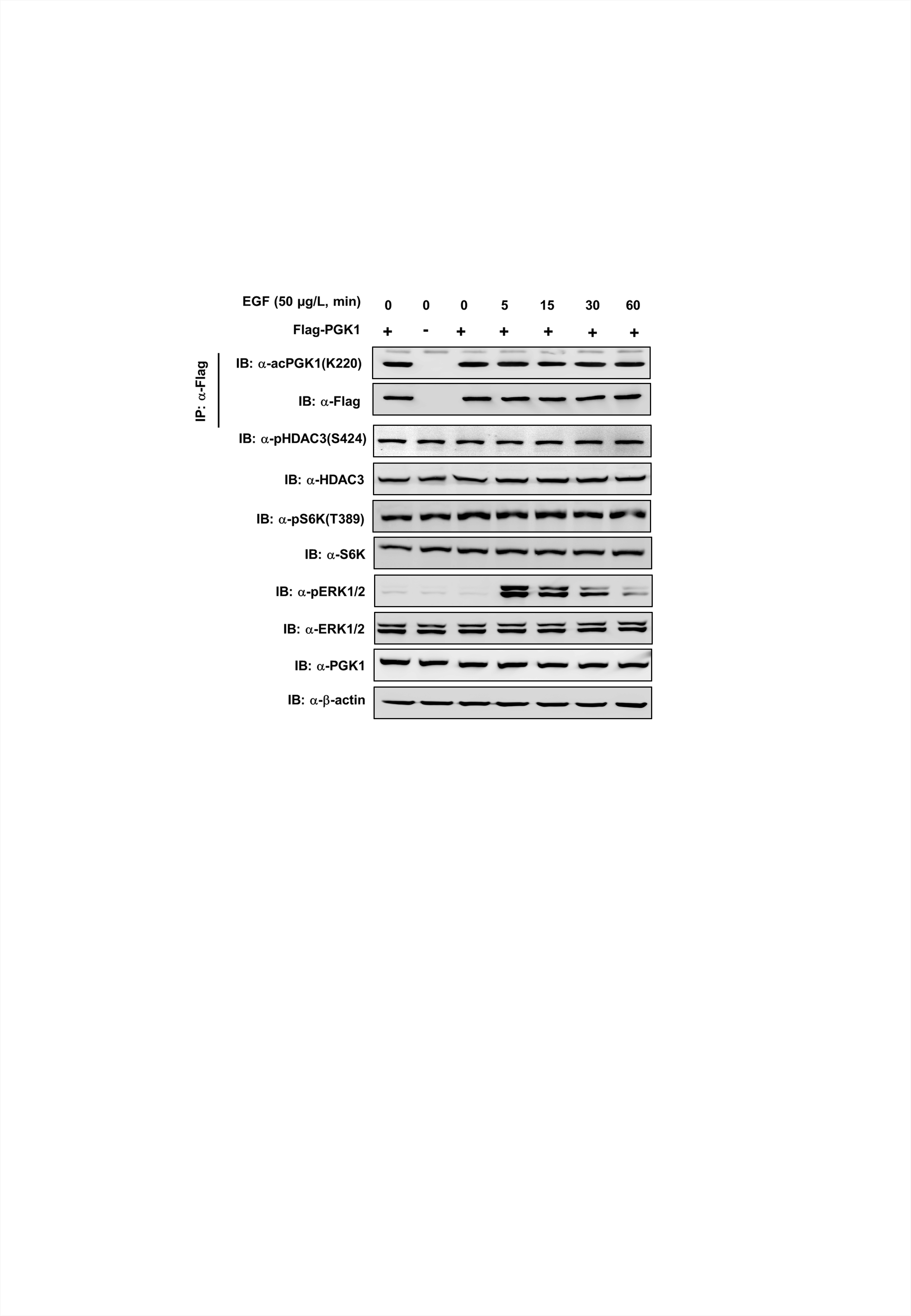

Supplement: S11 Fig — Flag-tagged PGK1 was ectopically expressed in HEK293T cells treated with EGF (50 μg/L) for the indicated time periods. Endogenous proteins of ERK1/2, S6K, HDAC3 and their phosphorylation were detected by western blotting. PGK1 proteins were purified by Flag beads, and PGK1 K220 acetylation was determined by western blot. (TIF) [file pbio.1002243.s012.tif]

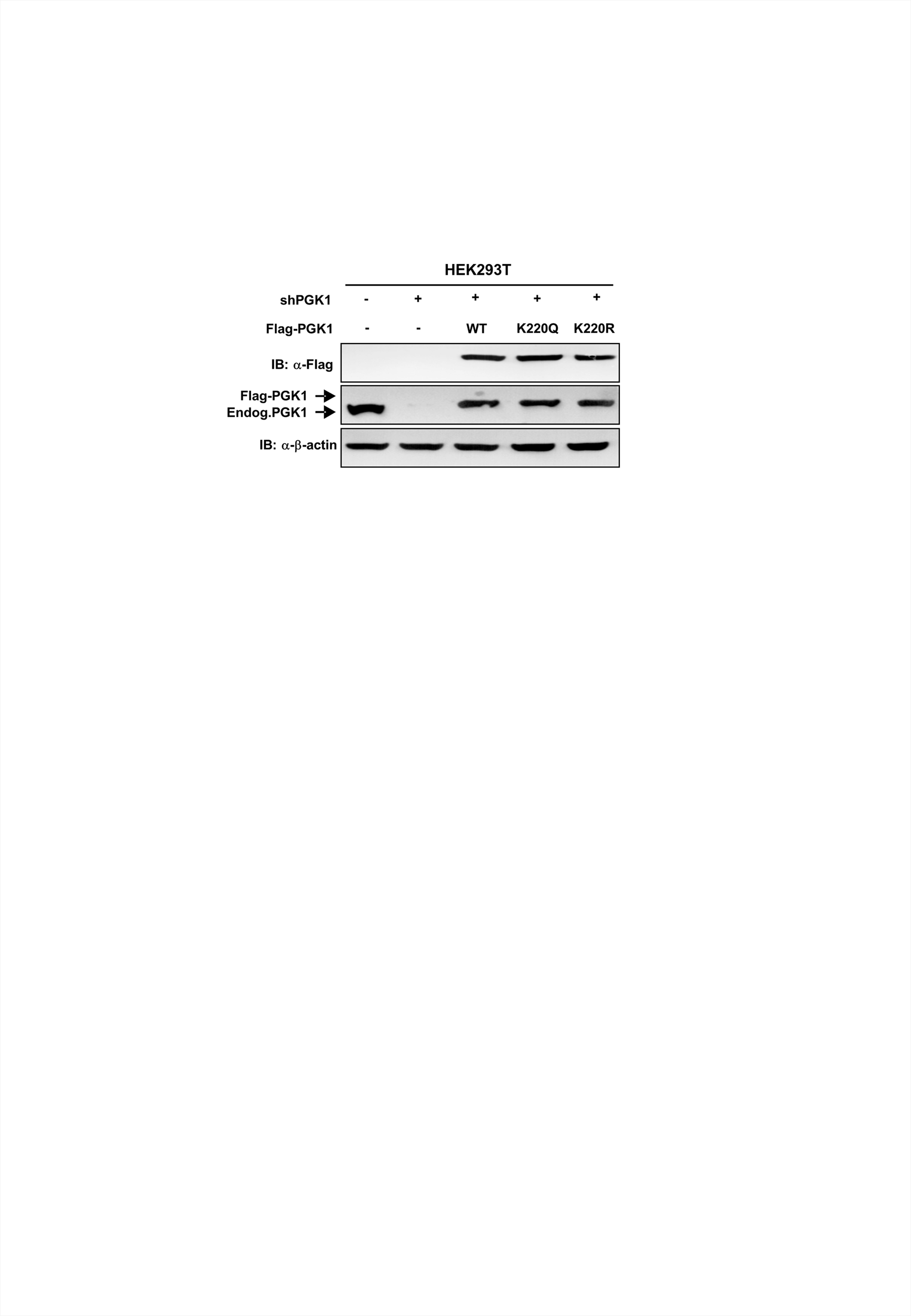

Supplement: S12 Fig — Stable HEK293T cells with knockdown of endogenous PGK1 were transfected with retroviral plasmids expressing the indicated proteins. PGK1 knockdown efficiency and re-expression were determined by western blot. (TIF) [file pbio.1002243.s013.tif]

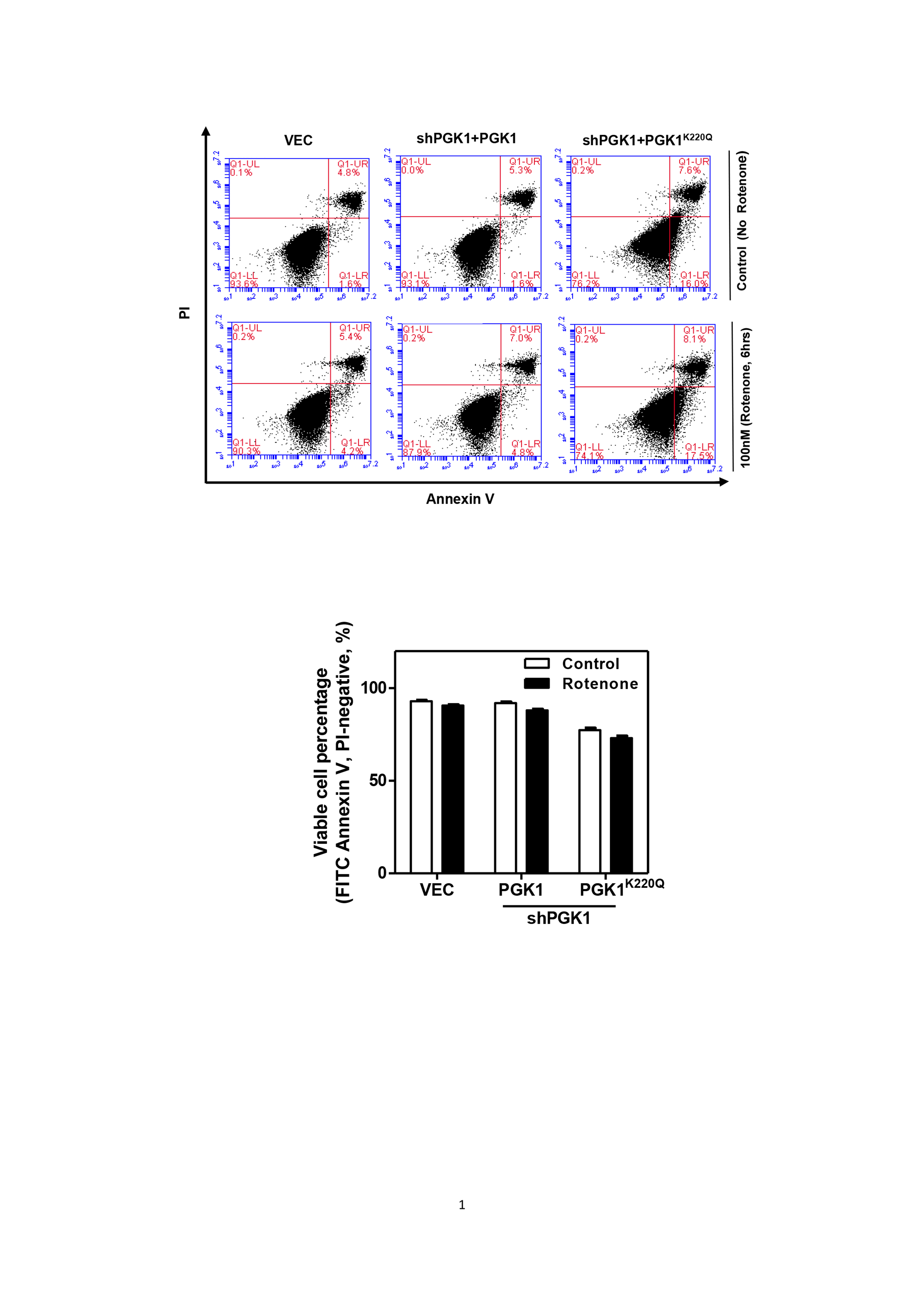

Supplement: S13 Fig — Stable HEK293T cells were treated with rotenone (100 nM) for 6 hr, and then the percentage of viable cells was determined by flow cytometric analysis using Annexin V and propidium iodode (PI) antibodies as described in S1 Text. Shown are average values with standard deviation (S.D.) of triplicated experiments. The numerical data and statistical analysis used in the figures are included in S1 Data. (TIF) [file pbio.1002243.s014.tif]

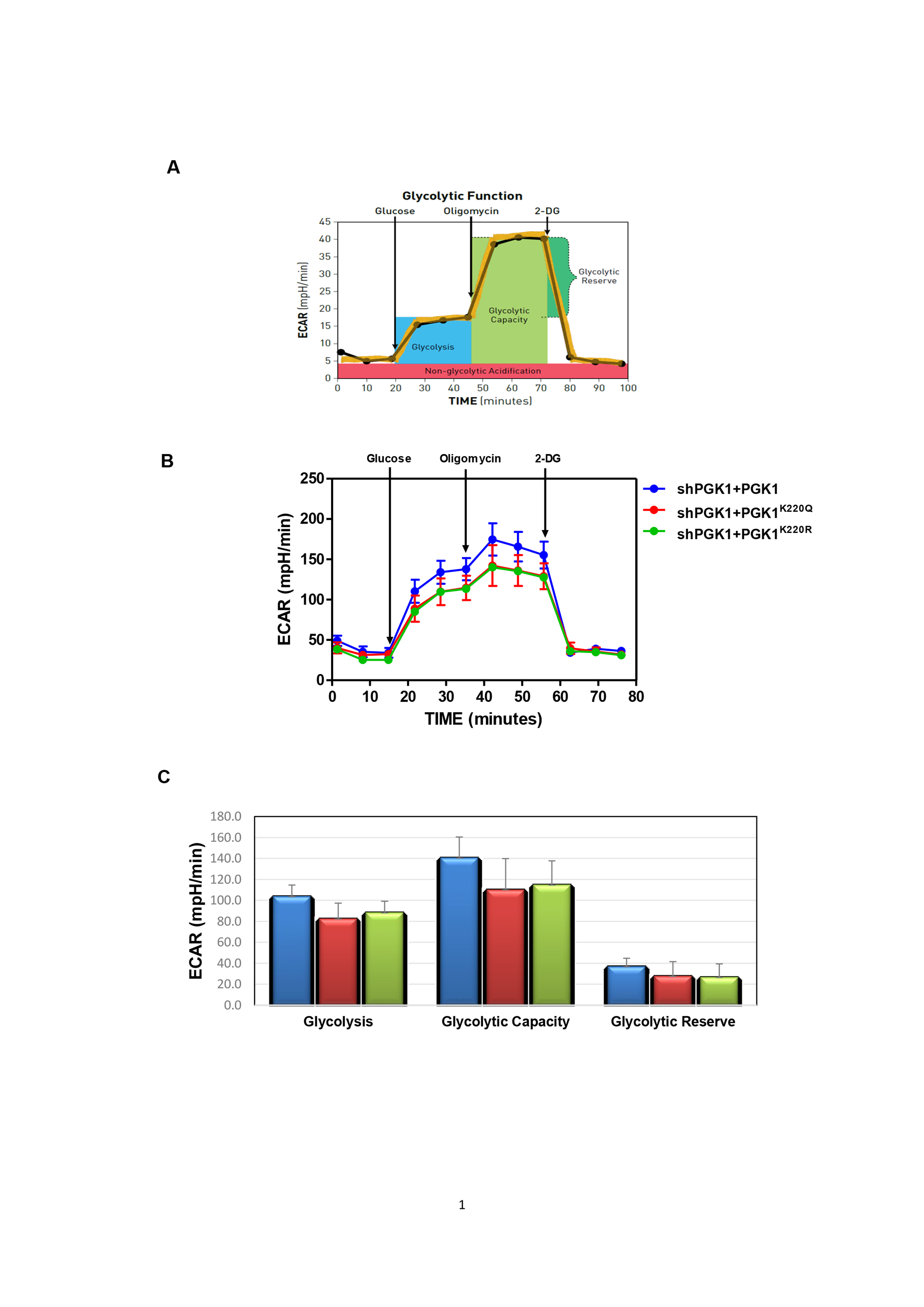

Supplement: S14 Fig — Extracellular acidification rate (ECAR) was determined in HEK293T cells with PGK1 knocking-down and putting-back using a Seahorse Bioscience XFe 96 analyzer system as described in S1 Text (as shown in A). The glycolytic function of rescued cells expressing wild-type PGK1 (blue circle), K220Q mutant PGK1 (red circle), and K220R mutant PGK1 (green circle) was compared (B). The parameters reflecting glycolysis, glycolytic capacity, and glycolytic reserve were determined by calculating Rate Measurement Equation (C). Shown are average values with standard error of the mean (S.E.M.) of triplicated experiments. The numerical data and statistical analysis used in the figures are included in S1 Data. (TIF) [file pbio.1002243.s015.tif]

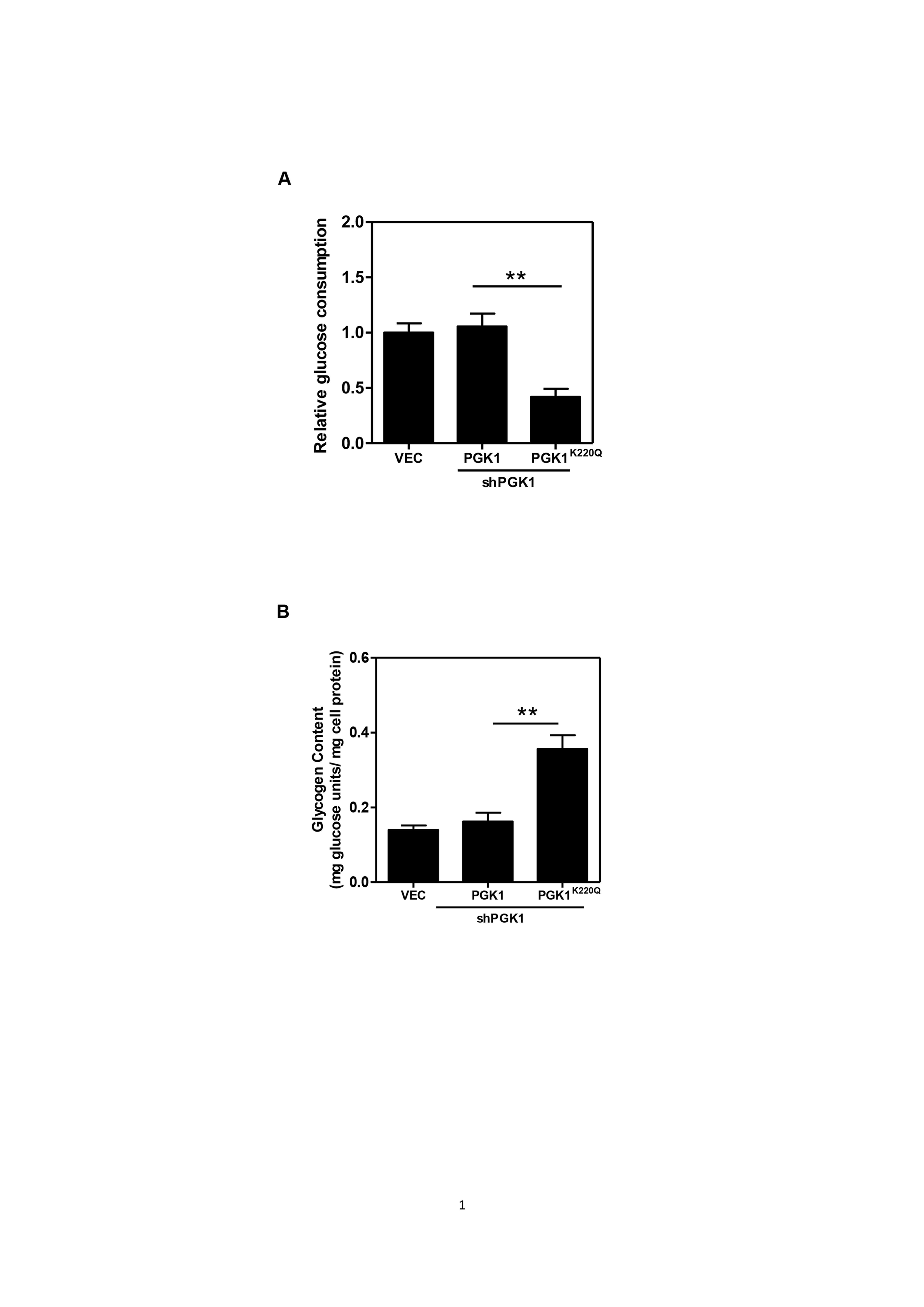

Supplement: S15 Fig — Relative glucose consumption (A) and glycogen storage levels (B) in the indicated stable HEK293T cells were measured as described in S1 Text. Shown are average values with standard deviation (S.D.) of triplicated experiments. ** denotes p < 0.01 for the indicated comparisons. The numerical data and statistical analysis used in the figures are included in S1 Data. (TIF) [file pbio.1002243.s016.tif]

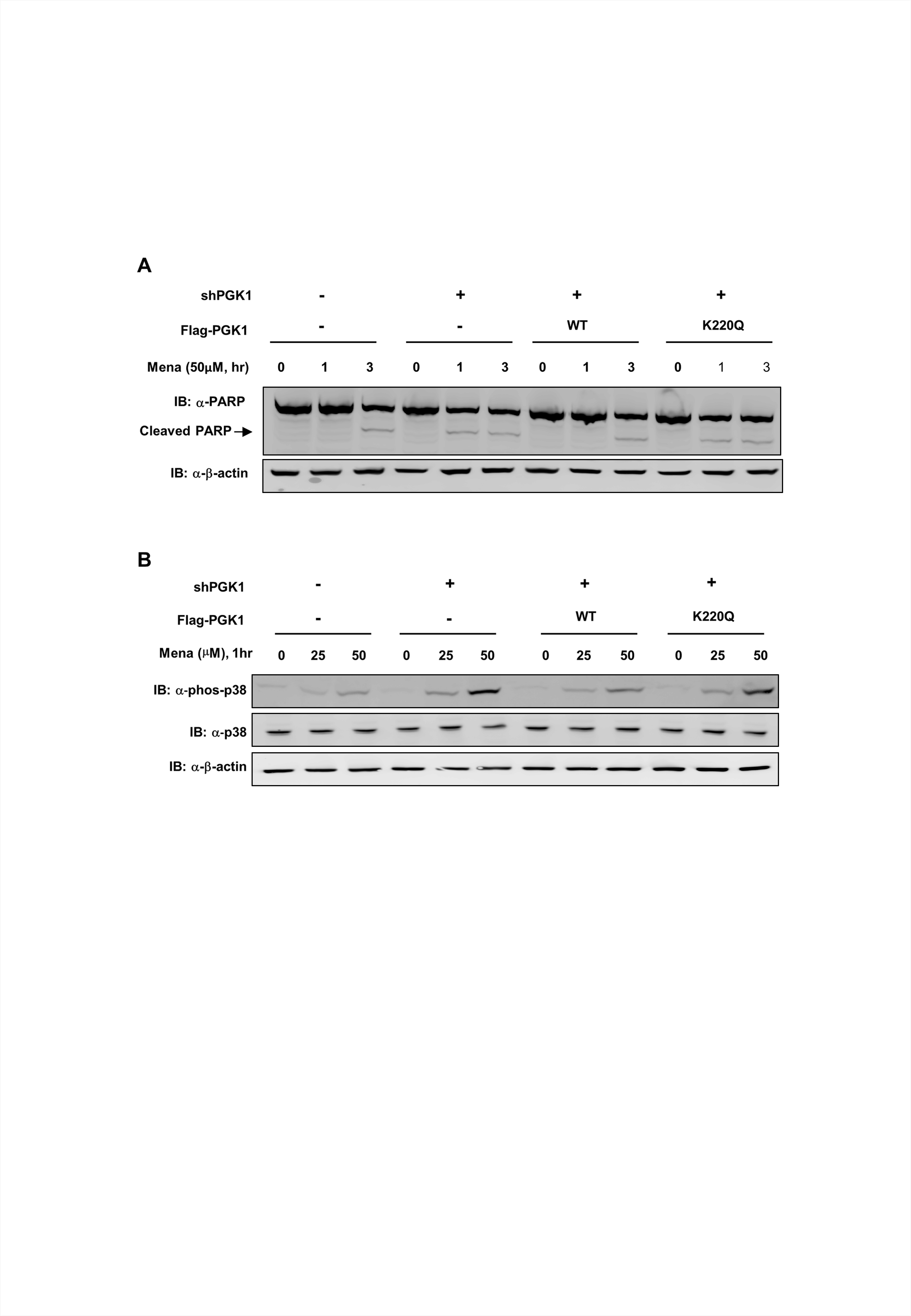

Supplement: S16 Fig — (A, B) Stable HEK293T cells were treated with menadione for the indicated concentrations and periods. The protein expression of PARP and p38 MAPK and their phosphorylation were determined by western blot analysis. (TIF) [file pbio.1002243.s017.tif]

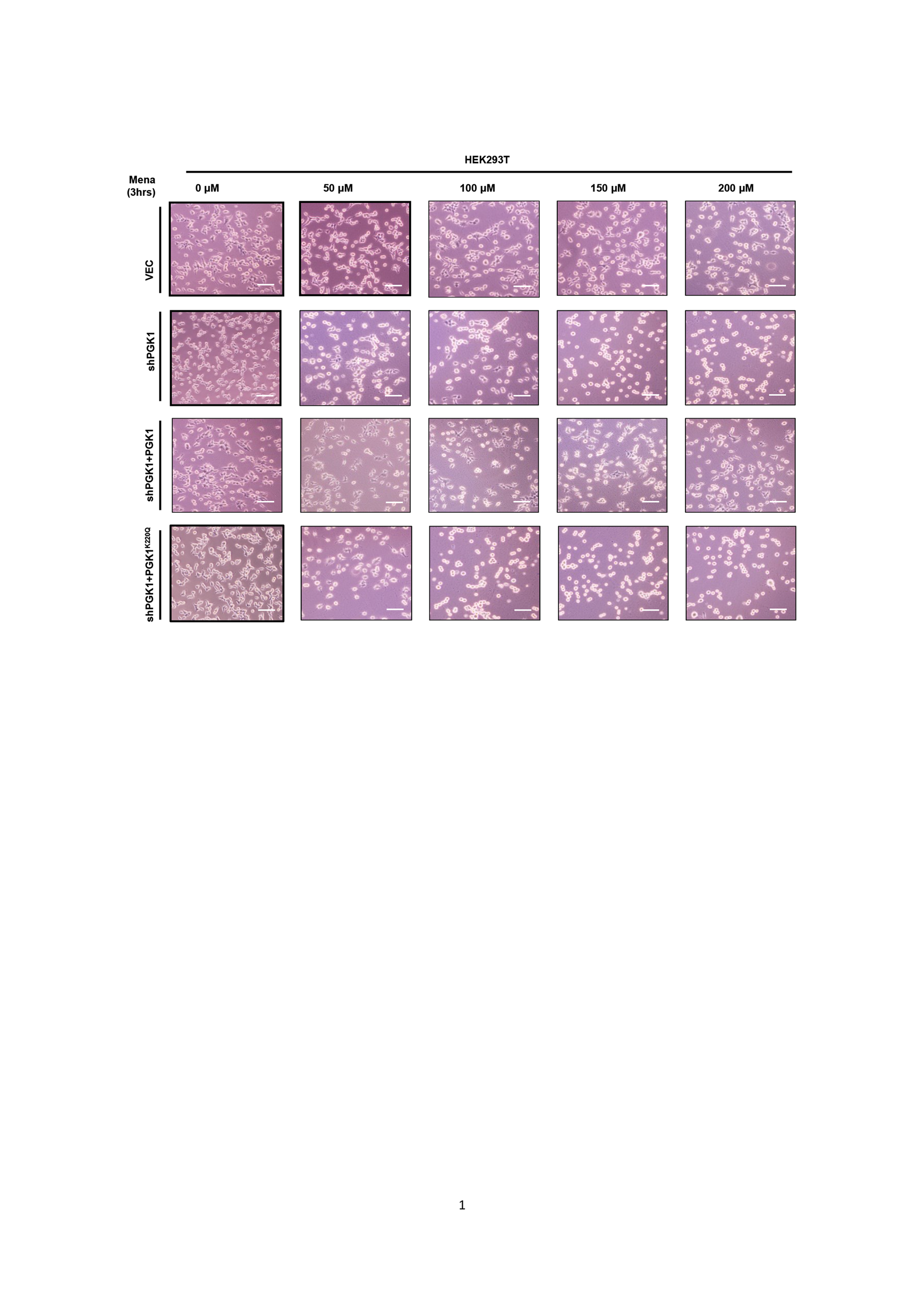

Supplement: S17 Fig — Stable HEK293T cells were treated with menadione of the indicated concentration for 3 hr. The changes in cell morphology were monitored under the microscope. Scale bars are 200 μm. (TIF) [file pbio.1002243.s018.tif]
