# Supplementary material for: Insulin and mTOR Pathway Regulate HDAC3-Mediated Deacetylation and Activation of PGK1
Source: PLoS Biol. 2015 Sep 10;13(9):e1002243. doi: 10.1371/journal.pbio.1002243 (PMC4565669; doi:10.1371/journal.pbio.1002243)
Supplement: S1 Table — (DOCX) [file pbio.1002243.s019.docx]

**Table S1. The list of oligonucleotides and primer sequences used in this study**

| **Name** | **Sequence(5'-3')** | **Note** |
| --- | --- | --- |
| **si*ATF2*-#1** | GGAGCCTTCTGTTGTAGAA | siRNA |
| **si*ATF2*-#2** | CCCAATGTGCTGCTTACAA | siRNA |
| **si*ATF2*-#3** | GCTATCATACTGCTGATAA | siRNA |
| **si*KAT1*-#1** | GCACAAACACGAATGATTT | siRNA |
| **si*KAT1*-#2** | CCATGGTGCTCAACTTCTT | siRNA |
| **si*KAT1*-#3** | GGAAGATTACCGGCGTGTT | siRNA |
| **si*KAT2A*-#1** | CCGAGTGGAGAAGGACAAA | siRNA |
| **si*KAT2A*-#2** | GCATGCCTAAGGAGTATAT | siRNA |
| **si*KAT2A*-#3** | TCTTCTACTTCAAGCTCAA | siRNA |
| **si*KAT2B*-#1** | GCAGATACCAAACAAGTTT | siRNA |
| **si*KAT2B*-#2** | GCATCCAAACAGTTATCAA | siRNA |
| **si*KAT2B*-#3** | CCGTATGTTCCCATCTCAA | siRNA |
| **si*KAT3A*-#1** | GCAGCTGGTTCTACTGCTT | siRNA |
| **si*KAT3A*-#2** | CCATTTCTCCTTCCCGAAT | siRNA |
| **si*KAT3A*-#3** | GGAAGCAGCTGTGTACCAT | siRNA |
| **si*KAT3B*-#1** | GCAGCTCAACCATCCACTA | siRNA |
| **si*KAT3B*-#2** | CCAGCCAGCTGTAAGCATT | siRNA |
| **si*KAT3B*-#3** | GCCAATTGCTCACTGCCAT | siRNA |
| **si*KAT4*-#1** | GCAGGTAACACAGGAAGGT | siRNA |
| **si*KAT4*-#2** | GGTGGGTATGAGGTATCAG | siRNA |
| **si*KAT4*-#3** | GAGGAGGACAGTGAGGATT | siRNA |
| **si*KAT5*-#1** | CCACAGGAACTCACCACAT | siRNA |
| **si*KAT5*-#2** | GCAATGAGATTTACCGCAA | siRNA |
| **si*KAT5*-#3** | GCTGATCGAGTTCAGCTAT | siRNA |
| **si*KAT6A*-#1** | GCATGTGGATATGTCAAAT | siRNA |
| **si*KAT6A*-#2** | GCTCTGAAGTGCAGATTAA | siRNA |
| **si*KAT6A*-#3** | CCTCACATCTCCTCTGCTT | siRNA |
| **si*KAT6B*-#1** | GGACGACACCTTTCAGGAT | siRNA |
| **si*KAT6B*-#2** | CCATCAGTTTCAGATCATT | siRNA |
| **si*KAT6B*-#3** | CCACCCATCAGTCACAAAT | siRNA |
| **si*KAT7*-#1** | GGAGAAGTTAAGGCTGCAA | siRNA |
| **si*KAT7*-#2** | CCACCTGGTGATGAGATAT | siRNA |
| **si*KAT7*-#3** | GCTGTCACCTGATTGGATA | siRNA |
| **si*KAT8*-#1** | GCAAGCATGATGAGATCAA | siRNA |
| **si*KAT8*-#2** | GCCTGGTATTTCTCACCAT | siRNA |
| **si*KAT8*-#3** | CCATAAGACACTGTACTTT | siRNA |
| **si*KAT9*-#1** | GCCAAACGTGGGACTAGAA | siRNA |
| **si*KAT9*-#2** | CCGAGTACAGAGGGATATT | siRNA |
| **si*KAT9*-#3** | CCGGGATCCTACTAAATTT | siRNA |
| **si*KAT12*-#1** | GCAGTTTCAGCTGCCGTTT | siRNA |
| **si*KAT12*-#2** | GCAAGAGGCTCTTATGTAT | siRNA |
| **si*KAT12*-#3** | GCACTGTAGTTTGTGTAAA | siRNA |
| **si*KAT13A*-#1** | CCAGCTACTTAGGTTACAA | siRNA |
| **si*KAT13A*-#2** | CCAGTTCACAGAGCAGTAA | siRNA |
| **si*KAT13A*-#3** | GGAAGCAATTGATAACCAA | siRNA |
| **si*KAT13B*-#1** | GCTGATATCTGCCAATCTT | siRNA |
| **si*KAT13B*-#2** | CCAGTAACATAGCTTCATT | siRNA |
| **si*KAT13B*-#3** | CCGGCAGAATGGAACCTAT | siRNA |
| **si*KAT13C*-#1** | GGAACAGCCATACCTTCAA | siRNA |
| **si*KAT13C*-#2** | GCAGTGAGCTGGACAACTT | siRNA |
| **si*KAT13C*-#3** | CCAGCCACTTATGAATCAA | siRNA |
| **si*KAT13D*-#1** | GCTGGAAAGTGACTCATTA | siRNA |
| **si*KAT13D*-#2** | GGAAATGTGTACTGTTGAA | siRNA |
| **si*KAT13D*-#3** | GGTACCAAGTACCATGCTT | siRNA |
| **si*KAT14*-#1** | GGAACAGCTCAGTTACCTT | siRNA |
| **si*KAT14*-#2** | GGAGCCAAAGAAGGAGGAA | siRNA |
| **si*KAT14*-#3** | GCTACTGTACCAGAAGTTT | siRNA |
| **ATF2-Forward** | GGTGCTTTGTAAACACGGCT | qRT-PCR |
| **ATF2-Reverse** | GCAGTCCTTTCTCAAGTTTCC | qRT-PCR |
| **KAT1-Forward** | AACACCAACACAGCAATTGAA | qRT-PCR |
| **KAT1-Reverse** | CATCCCCAAAGAGTTGATGG | qRT-PCR |
| **KAT2A-Forward** | GTGCTGTCACCTCGAATGAG | qRT-PCR |
| **KAT2A-Reverse** | CGGCGTAGGTGAGGAAGTAG | qRT-PCR |
| **KAT2B-Forward** | GGCCAAGAAACTGGAGAAACT | qRT-PCR |
| **KAT2B-Reverse** | GGTGAGGGGTTAGGGTTTTT | qRT-PCR |
| **KAT3A-Forward** | TGAGACCCTAACGCAGGTTT | qRT-PCR |
| **KAT3A-Reverse** | CAAATGGACTTGTGTTCCCA | qRT-PCR |
| **KAT3B-Forward** | GCGGCCTAAACTCTCATCTC | qRT-PCR |
| **KAT3B-Reverse** | TCTGGTAAGTCGTGCTCCAA | qRT-PCR |
| **KAT4-Forward** | AGAGTCGGGAGAGCTTTCTG | qRT-PCR |
| **KAT4-Reverse** | CACAATCTCCTGGGCAGTCT | qRT-PCR |
| **KAT5-Forward** | CATCCTCCAGGCAATGAGAT | qRT-PCR |
| **KAT5-Reverse** | CTTGGCCAAAAGACACAGGT | qRT-PCR |
| **KAT6A-Forward** | CGTGGATGGGAAAGAGAGTT | qRT-PCR |
| **KAT6A-Reverse** | ACAGATGGGGATTGGTTCAG | qRT-PCR |
| **KAT6B-Forward** | AAAGGGGCACCTCAGTATCC | qRT-PCR |
| **KAT6B-Reverse** | CATATTGGAATGGGATCAGCA | qRT-PCR |
| **KAT7-Forward** | TGAAGTGTCCTACACCAGGC | qRT-PCR |
| **KAT7-Reverse** | TGATACAGTGGGCATCCTGA | qRT-PCR |
| **KAT8-Forward** | GATCACTCGCAACCAAAAGC | qRT-PCR |
| **KAT8-Reverse** | CCTCATGCTCCTTCTCCAAG | qRT-PCR |
| **KAT9-Forward** | GAAGAACATGGGTCTGGGAA | qRT-PCR |
| **KAT9-Reverse** | CGGGCCTTGTAATCTGTAGC | qRT-PCR |
| **KAT12-Forward** | GCTCAACAGCTGTCTCCTCA | qRT-PCR |
| **KAT12-Reverse** | AAAGTCTGACTGACCGTGGG | qRT-PCR |
| **KAT13A-Forward** | TCTGAGGGGCTTAGAAATTAACA | qRT-PCR |
| **KAT13A-Reverse** | TGATGTTTTCCAGAGATGGCT | qRT-PCR |
| **KAT13B-Forward** | CCGATTTAAAGCTGAGCTGC | qRT-PCR |
| **KAT13B-Reverse** | CAGTCAAAGGATGTTCAAGCA | qRT-PCR |
| **KAT13C-Forward** | CACCTGACGGCGTGACC | qRT-PCR |
| **KAT13C-Reverse** | TCAGCAACTGTGCCTGTAAAC | qRT-PCR |
| **KAT13D-Forward** | GAAGGAAATCTGGCCGC | qRT-PCR |
| **KAT13D-Reverse** | TCCAAAGGCATCTTACAATGTG | qRT-PCR |
| **KAT14-Forward** | CTGGACAAGCCACGTACAGA | qRT-PCR |
| **KAT14-Reverse** | TCTGGTGTCGAAATCCCTTC | qRT-PCR |
