# Supplementary material for: Insulin and mTOR Pathway Regulate HDAC3-Mediated Deacetylation and Activation of PGK1
Source: PLoS Biol. 2015 Sep 10;13(9):e1002243. doi: 10.1371/journal.pbio.1002243 (PMC4565669; doi:10.1371/journal.pbio.1002243)
Supplement: S1 Text — For more details about antibodies, plasmids, cell culture and treatment, immunoprecipitation and western blotting, RNA isolation and quantitative real-time PCR, animal experiments, RNA interference, protein concentration determination, protein expression and purification, pull-down assay, in vitro deacetylation assay, HDAC3 activity assay, measurement of intracellular ATP levels, flow cytometry, liquid chromatography mass spectrometry (LC-MS), extracellular acidification rate (ECAR), glucose depletion assay, glycogen content measurement, measurement of intracellular ROS levels, or cell survival assay, please refer to S1 Text. (DOCX) [file pbio.1002243.s020.docx]

**S1 Text. Supplementary Materials and Methods**

**Antibodies**

Antibodies to PGK1 (Santa Cruz), HDAC3 (Santa Cruz; Epitomics) HDAC3 (phospho S424) (Abcam; Cell signaling), AKT1 (Cell signaling), AKT1 (phosphor S473) (Cell signaling), S6K (Cell signaling), S6K (phospho T389) (Cell signaling), ERK1/2 (Cell signaling), ERK1/2 (phospho T202/Y204) (Cell signaling), PARP (Cell Signalling), p38 MAPK (Cell Signalling), p38 MAPK phosphorylation (Cell Signalling), Flag (Shanghai Genomics), HA (Santa Cruz) were purchased commercially.

**Plasmids**

The cDNA encoding full-length human PGK1, HDAC1-7 were cloned into Flag, HA, or His-tagged vectors (pRK7-N-Flag; pcDNA3-HA, pSJ3). Point mutations of PGK1 and HDAC3 were generated by site-directed mutagenesis employing a QuickChange Site-Directed Mutagenesis kit (Stratagene). The full-length human PGK1 and mutants were sub-cloned into His-pSJ3. All expression constructs were verified by DNA sequencing.

**Cell culture and treatment**

HEK293T cells were cultured in Dulbecco’s modified Eagle’s medium (DMEM) (Invitrogen) supplemented with 10% new born bovine serum (Biochrom Germany) in the presence of penicillin, streptomycin, and 8 mM L-glutamine (Invitrogen). Stable cells were cultured in DMEM supplemented with 10% fetal bovine serum (Biochrom, Germany) in the presence of penicillin, streptomycin. Cells were incubated in DMEM containing 0.5% fetal bovine serum for 12 hrs before treatment with insulin or treated with 50 μg/L EGF (PeproTeCh) after 12 hrs starvation. Other agents, such as menadione (Sigma), rotenone (Sigma), LY294002 (Selleckchem), Wortmannin (Sigma), Rapamycin (Santa Cruz), and MK-2206 2HCL (Selleckchem) were added into the culture medium at the indicated concentrations.

Plasmid transfection was carried out using calcium phosphate method or lipofectamine 2000 (Invitrogen). The deacetylase inhibitor Tricostatin A (TSA, 5 μM) and Nicotinamide (NAM, 10 mM) were added to the culture medium 12 hrs and 4 hrs before cell harvest, respectively.

**Immunoprecipitation and western blotting**

Cells were lysed in ice-cold NP40 buffer containing 50 mM Tris-HCl (pH 7.5), 150 mM NaCl, 0.3% Nonidet P-40, 1 μg/ml Aprotinin, 1 μg/ml Leupeptin, 1 μg/ml Pepstatin, 1 mM Na_3_VO_4_, 10mM NaF, and 1 mM PMSF. Cell lysate were incubated with Flag beads (Sigma) for 3 hrs at 4 ºC, or with appropriate antibody for 1 hr, followed by incubating with Protein-A beads (Upstate) for another 2 hrs at 4°C. After washing three times with ice-cold NP-40 buffer, the proteins were either eluted by Flag peptides (Gilson Biochemical) for activity assay or denatured by SDS loading buffer containing DTT before western-blot analysis. Flag, HA and Myc western blotting was blocked with 5% fat-free milk (BD Biosciences), and standard procedures were employed.

For acetylation western blotting, 50 mM Tris (pH 7.5) with 10% (v/v) Tween 20 and 1% peptone (AMRESCO) was used for blocking, and 50 mM Tris (pH 7.5) with 0.1% peptone was used to prepare primary and secondary antibodies.

**RNA isolation and quantitative real-time PCR**

Total RNA was extracted from cultured cells using Trizol reagent (Invitrogen). RNA was reverse transcribed with random primers, and quantitative real time-PCR was performed following the manufacturer’s instruction using an Applied Biosystems 7500 Sequence Detection System with SYBR green labeling (Takara). *β-actin* was used as a endogenous controls. Primer sequences were present in the supplementary Table S1.

**RNA interference**

*HDAC3* knockdown was carried out by using synthetic siRNA oligonucleotides synthesized by Genepharma Inc. Shanghai. siRNAs were transfected into the culture cells by Lipofectamin 2000 (Invitrogen). The cells were harvested at 36-48 hrs after transfection. Three effective target sequences to exclude off-target effects were employed. The siRNA sequences were as follows:

*HDAC3* siRNA-1: 5’- GCCGGUUAUCAACCAGGUA-3’

*HDAC3* siRNA-2: 5’- AAUAUCCCUCUACUCGUGCUG-3’

*HDAC3* siRNA-3: 5’- CCGCCAGACAAUCUUUGAA-3’

**Protein concentration determination**

Protein concentrations were determined by a Bio-Rad Protein Assay Kit (Bio-Rad). Relative protein concentrations were quantified by measuring the band intensity of western blots by using Amersham Bisciences ImageQuant TL 2005v software.

**Protein expression and purification**

GST-HDAC3, wild-type and K220 mutant His-PGK1 proteins were overexpressed in BL21 (DE3). The transformants were grown at 37 °C to an OD600 of LB medium and then incubated with 1 mM isopropyl-b-D-thiogalactopyranoside for 16 h at 15 °C. Cells were resuspended in lysis buffer [25 mM HEPES (pH 8.0), 150 mM NaCl, protease inhibitors (Roche)], and then the lysate was cleared by centrifugation at 40,000 g for 1 hr. The supernatants of cell lysate were purified onto Ni and GST affinity columns (GE healthcare) respectively. After washing with a 20 mM imidazole-containing buffer, the fusion proteins were eluted [25 mM HEPES (pH8.0), 150 mM NaCl and 250 mM imidazole] and further purified by gel filtration (Superdex 200, GE healthcare). Purity of recombinant protein was checked by 10% SDS-PAGE for further analysis.

**In vitro pull-down assay**

30 μg purified recombinant GST-HDAC3 protein and 10 μg purified recombinant His-PGK1 protein were mixed in the binding buffer (150 mM NaCl, 0.1% TritonX-100, 20 mM Tris-HCl at pH 8.0, 5% glycerol) at 4 ºC for 2 hrs. His-PGK proteins were then immobilized to 20 μl of Ni beads (QIAGEN) for 1 hour at 4 °C. After washing for three times with washing buffer (50 mM NaCl, 0.1% TritonX-100, 20 mM Tris-HCl at pH 8.0, 5% glycerol) at 2000 rpm for 1 min, bound proteins were subjected to SDS-PAGE and stained by Coomassie blue.

**In vitro deacetylation assay**

HEK293T cells were transfected with Flag-tagged PGK1, and cell extracts were immunoprecipitated with Flag beads for 3 hrs at 4 ºC. Then, the immunoprecipitated PGK1 was incubated with or without 30μg GST-HDAC3 in the reaction buffer (20 mM Tris at pH 8.0, 150 mM NaCl, 1 mM Zinc and 10% glycerol) at 37 °C for 1 hr in the presence or absence of TSA (5 μM). After incubation, PGK1 acetylation and enzyme activity were determined.

**HDAC3 activity assay**

HDAC3 activity was determined by using a commercial HDAC3 enzyme activity assay kit (Sigma). Briefly, immunoprecipitated samples were incubated with 2 μl HDAC3 substrate [R-H-K-K(Ac)-AFC] and 23 μl HDAC3 assay buffer at 37°C for 15 min. Once incubation is completed, 10 μl developer and 40 μl HDAC3 assay buffer were added and then incubated at 37°C for another 5 min. The released quenched fluorescent group, AFC, which can be detected at Em/Ex=380/500 by a Fluorescence multiwall plate reader (BioTek).

**Measurement of intracellular ATP levels**

Cellular ATP levels were determined by using a commercial Firefly-Luciferase ATP assay kit (Beyotime). Briefly, cells were treated with 100 nM rotenone (Sigma) for 6 hrs and then lysed with ATP-releasing buffer. After centrifugation for 10 min at 12000 rpm, the supernatant was incubated in the ATP-detection buffer containing Firefly Luciferin and Firely Luciferase enzyme reagent for 5 min, and then measured RLU by Modulus^TM^ Microplate Reader (Turner Biosystems). The ATP levels were normalized against protein concentrations by using a Bio-Rad Protein Assay Kit (Bio-Rad).

**Flow cytometry**

Cell apoptosis was determined by using a commercial FITC Annexin Ⅴ Apoptosis Detection Kit (BD Pharmingen). Briefly, cells were washed twice with cold PBS and then resuspended in buffer at a concentration of 1×10^6^ cells/ml. 5 μl of FITC Annexin V and 5 μl propidium iodode (PI) were added into the buffer and cells were then incubated at 25°C for 15 min in the dark. The percentages of Annexin V and/or PI-positive cells were determined by flow cytometer (BD Accuri^TM^ C6).

**Liquid chromatography mass spectrometry (LC-MS)**

Intracellular levels of glycolytic metabolites were determined by LC-MS. Briefly, stable HEK293T cells were cultured in DMEM containing 10% dialyzed fetal bovine serum (Gibco). Cells were harvested by using 80% (v/v) pre-cold (-80°C) methanol added 5 μm glutaric acid as external standard to correct the experimental error. The supernatant was centrifuged at 4 °C at 14000 rpm for 30 min. After filtration, 10 μl supernatant was subjected to LC-MS analysis using 5500 QTRAP hybrid dual quadrupole ion trap mass spectrometer (AB/SCIEX) for monitoring the 3-PG (Q1 185.00, Q3 97), Ser ( Q1 106.00, Q3 60). A Luna NH2 HPLC column (5.0 μm; 4.6 mm i.d. × 50 mm length; Phenomenex, cat.no. 00B-4378-E0) were used. Ionization was attained in the negative mode and positive mode. The LC mobile phase are Solvent A: pH = 9.0: 95% (vol/vol) water, 5% (vol/vol) acetonitrile, 20 mM ammonium hydroxide, 20 mM ammonium acetate; Solvent B: 100% acetonitrile. The gradients are as follows: t=0.0, 85% B; t=3.0min, 85% B to 30% B; t=12.0min, 30% B to 2% B; t=15.0min, 2% B; t=16.0min, 2% B to 85% B; t=23.0 min, 85% B. The flow rate is 200 μl min^−1^. Peak detection and integration was performed with Analyst Software (AB SCIEX). Cell numbers and glutaric acid were used for sample normalization.

**Extracelluar acidification rate (ECAR)**

ECAR was determined using a Seahorse XF96 instrument (Seahorse Biosciences) according to the manufacturer’s instructions. In brief, a Seahorse 96-well plate was pre-coated with 0.1 mg/ml poly-D-lysine hydrobromide (Sigma). Stable HEK293T cells were counted (Countstar IC1000) and seeded into the plate at a cell density of 2,0000 cell/well, following culture for 16 hrs. After that, 10 mM glucose, 2 μM oligomycin, and 2DG were added into different ports of the Seahorse cartridge. Each experimental group was analyzed using three replicates. Parameters which reflect the glycolytic function, including glycolysis, glycolytic capacity, and glycolytic reserve, were calculated by Rate Measurement Equation as following:

Glycolysis: (Maximum rate measurement before oligomycin injection) – (Last rate measurement before glucose injection)

Glycolytic Capacity: (Maximum rate measurement after oligomycin injection) – (Last rate measurement before glucose injection)

Glycolytic Reserve: (Glycolytic Capacity) – (Glycolysis)

**Glucose depletion assay**

Glucose consumption was determined by using a colorimetric glucose assay kit (GAGO20; Sigma-Aldrich). Briefly, medium was collected after 3 hrs of cell culture. After incubating the medium with reagent assay at 37°C for 30 min, the absorbance at 540 nm was measured using a UV/Visible spectrophotometer reader. The depletion of glucose was determined by subtracting the final medium glucose concentration from initial medium glucose concentration in the culture medium. Data were normalized against cell number countered by using a hemocytometer (ShanghaiQiujing).

**Glycogen content measurement**

Cells were lysed with supersonic in 1ml PBS (pH4.8). Cell lysate was heated for 10 min to liberate stored glycogen and to inactivate enzymes, which may produce extra glucose. 4U amyloglucosidase (Sigma) was added into the supernatant after centrifugation for 15 min at 13,000 rpm. The cell lysate without amyloglucosidase was included as a control. After incubating the mixture at 50°C for 1 hr and following by boiling for 10 min at 99°C, the glycogen content was colorimetrically measured using a glucose assay kit (GAGO20, Sigma). The glycogen content were normalized against protein concentrations by using a Bio-Rad Protein Assay Kit (Bio-Rad).

**Measurement of intracellular ROS levels**

Intracellular ROS levels were determined by using a fluorescent dye 2’, 7’-dichlorofluorescein diacetate (H_2_DCF-DA, Sigma). Briefly, cells were washed with PBS and incubated with 10 μM H_2_DCF-DA at 37°C for 30 min to load the fluorescent dye. Cells were washed twice with PBS and trypsinized for menadione treatments. Fluorescence (Ex.488 nm, Em.525 nm) was monitored by a SpectraMax M5 Microplate Reader (Molecular Devices).

**Cell survival assay**

A density of 4×10^4^ per well cells was seeded in six-well plates. Cells were treated with increased concentrations of menadione (Sigma). After incubation for 3 hrs, the remaining adhered and living cells were typsinized and counted by hemocytometer (ShanghaiQiujing).
